# Formation of chimeric genes with essential functions at the origin of eukaryotes

**DOI:** 10.1186/s12915-018-0500-0

**Published:** 2018-03-13

**Authors:** Raphaël Méheust, Debashish Bhattacharya, Jananan S. Pathmanathan, James O. McInerney, Philippe Lopez, Eric Bapteste

**Affiliations:** 1 Sorbonne Universités, UPMC Univ Paris 06, CNRS, Evolution Paris Seine - Institut de Biologie Paris Seine (EPS - IBPS), 75005 Paris, France; 2 Department of Biochemistry and Microbiology, Rutgers University, New Brunswick, NJ 08901 USA; 3 Division of Evolution and Genomic Sciences, School of Biological Sciences, Faculty of Biology, Medicine and Health, The University of Manchester, Manchester Academic Health Science Centre, M13 9PL, Manchester, UK

**Keywords:** Eukaryogenesis, Evolutionary transition, Chimeric genes, Evolutionary genomics, Endosymbiosis

## Abstract

**Background:**

Eukaryotes evolved from the symbiotic association of at least two prokaryotic partners, and a good deal is known about the timings, mechanisms, and dynamics of these evolutionary steps. Recently, it was shown that a new class of nuclear genes, symbiogenetic genes (S-genes), was formed concomitant with endosymbiosis and the subsequent evolution of eukaryotic photosynthetic lineages. Understanding their origins and contributions to eukaryogenesis would provide insights into the ways in which cellular complexity has evolved.

**Results:**

Here, we show that chimeric nuclear genes (S-genes), built from prokaryotic domains, are critical for explaining the leap forward in cellular complexity achieved during eukaryogenesis. A total of 282 S-gene families contributed solutions to many of the challenges faced by early eukaryotes, including enhancing the informational machinery, processing spliceosomal introns, tackling genotoxicity within the cell, and ensuring functional protein interactions in a larger, more compartmentalized cell. For hundreds of S-genes, we confirmed the origins of their components (bacterial, archaeal, or generally prokaryotic) by maximum likelihood phylogenies. Remarkably, Bacteria contributed nine-fold more S-genes than Archaea, including a two-fold greater contribution to informational functions. Therefore, there is an additional, large bacterial contribution to the evolution of eukaryotes, implying that fundamental eukaryotic properties do not strictly follow the traditional informational/operational divide for archaeal/bacterial contributions to eukaryogenesis.

**Conclusion:**

This study demonstrates the extent and process through which prokaryotic fragments from bacterial and archaeal genes inherited during eukaryogenesis underly the creation of novel chimeric genes with important functions.

**Electronic supplementary material:**

The online version of this article (10.1186/s12915-018-0500-0) contains supplementary material, which is available to authorized users.

## Background

It has recently been demonstrated that endosymbiosis and the subsequent evolution of eukaryotic photosynthetic lineages was concomitant with the formation of a novel class of nuclear genes, referred to as symbiogenetic genes (S-genes) [1]. New genes can evolve in many ways [2], including by duplication [3], de novo formation [4], or by the fusion of gene fragments that encode functional domains and give rise to novel chimeric proteins [5]. S-genes are in the latter category, and emerged in photosynthetic eukaryotes from the union of domains acquired by endosymbiotic gene transfer (EGT) from the plastid to the host nucleus, with domains of other origins. S-genes identified in algae and plants are primarily involved in the integration of an oxygen-evolving, potentially toxic endosymbiont in the eukaryotic host. Specifically, recycled genetic domains from plastid DNA contributed to the enhancement of metabolic integration and reactive oxygen species (ROS) detoxification in photosynthetic eukaryotes [1].

However, plastids are neither the first nor the only organelles present in eukaryotes [6]. Mitochondrial acquisition occurred earlier, likely driving eukaryogenesis. This major evolutionary transition [7, 8] took place about two billion years ago and involved two prokaryotic partners, one ancestral archaeum [9, 10] and one ancestral alpha-proteobacterium [11, 12]. Even though the details of the genetic, physiological, and structural basis of their merger remain to be established [13, 14], there is a consensus forming that eukaryotes are a genetic chimera because they are comprised of at least two genomes, namely a nuclear genome and DNA derived from one or two endosymbionts (i.e., the mitochondrion and plastid) [11].

During the evolution of eukaryotes, the mitochondrial genome has been significantly reduced in size, with many genes being lost and others being transferred, either intact or in pieces, to the host eukaryotic nucleus through EGT [15–17]. In addition to these EGT-derived genes, a recent analysis reported the presence of bacterial genes of non-alpha-proteobacterial provenance in the Last Eukaryotic Common Ancestor (LECA). This finding raises the possibility of additional bacterial contributions to the emergence of eukaryotes [18]. Regardless of the number of prokaryotic donors, the nuclear genome of eukaryotes encodes genes inherited both from Bacteria and Archaea. The bacterial sequences primarily encode operational functions, whereas genes of archaeal origin are usually involved in informational functions [19]. In addition to these ancestral genes of symbiotic origin, eukaryotes also contain lineage-specific genes [20, 21] created during, and after, eukaryogenesis. As a result, numerous eukaryotic features and processes (e.g., the nucleolus, the cytoskeleton, the DNA replication and transcription systems), while inherited from prokaryotes [22, 23], were ‘tinkered’ with and made more complex [24, 25] via the addition of essential components that lack prokaryotic homologs [25, 26]. Furthermore, eukaryotes have also evolved novel features (e.g., endoplasmic reticulum, Golgi, peroxisomes, spliceosome) without direct prokaryotic antecedents [27]. These innovations occurred early during eukaryogenesis because LECA was endowed with most of the structural traits present in extant lineages [25–28].

Despite this general knowledge regarding eukaryogenesis, the origin of many nuclear genes remains poorly understood. For example, one study reported that 63% of eukaryote nuclear genes lack homologs outside of eukaryotes [21]. In contrast, eukaryotic nuclear genes usually harbor multiple domains, which indicates that their evolution can follow multiple complex paths, including the fusion and fission of domains. Because the nuclear genome of eukaryotes hosts genes from multiple origins (with a pool of genes originating from Archaea and another from Bacteria), and because the taxonomic distribution of many of these genes indicates they evolved during eukaryogenesis, it is important (and still unknown) to elucidate their provenance. More specifically, did these genes arise via the remodeling of genetic material from distinct prokaryotic contributors? We predicted that aspects of the leap forward in organizational and compositional complexity from a consortium of prokaryotes resulted from the evolution of S-genes during the early stages of eukaryogenesis. Phylogenetic methods that use simultaneous alignment of collinear proteins sharing significant sequence similarity over all, or most, of their lengths are useful to analyze the contribution of transferred intact genes to eukaryote evolution. However, the detection of reticulate sequence evolution, such as the fusion and recycling of domains derived from heterologous proteins, benefits from alternative network approaches. Here, we have used sequence similarity networks [29] that rely on reconstruction of both full and partial (i.e., protein domain) sequence relationships using pairwise protein similarity values to determine whether S-genes played a critical role in eukaryogenesis.

We report the formation of S-genes (282 gene families) early in eukaryotic evolution. These chimerical proteins contributed essential components to macromolecular eukaryotic complexes, such as the ubiquitin system, the spliceosome, the SSU-processome, and the transcription and translation systems, and were involved in membrane trafficking and lipid metabolism. Remarkably, in eukaryotic informational genes, we detected twice as many S-genes of bacterial than of archaeal origin, in agreement with Cotton and McInerney [21]. Fundamental eukaryotic properties are thus derived from pieces of prokaryotic genes that have recombined with other domains. Early in their history, and thereafter, eukaryotes exploited domains from multiple co-interacting genomes to retool their own functional repertoire. This observation lies outside of the traditional informational versus operational divide of genetic contributions of archaeal and bacterial lineages, respectively, to the origin of eukaryote gene inventories.

## Results and discussion

### Early creation of S-genes

#### Detection of S-genes

We searched for homologous relationships between 614,589 proteins from 38 protists sampled from across eukaryotic diversity and 1,151,256 proteins from 382 prokaryotes. Briefly, we compared all sequences by BLAST [30], using sequence similarity to generate clusters (i.e., homologs that can be aligned over 80% of their length, see Methods) that were considered as gene families. This protocol led to 6733 clusters containing sequences from at least three eukaryotic taxa. We considered that a family was multidomain and composite (Additional file 1: Figure S1) when more than 50% of sequences from the family encoded at least two domains using CDD [31] or Pfam [32], and when FusedTriplets [29] indicated chimerism (Additional file 2: Figure S2). This conservative protocol returned 1621 composite multidomain gene families. We classified these families into three groups, based on the homology (or lack thereof) of composite eukaryotic sequences with prokaryotic sequences from a reference dataset of 2704 complete prokaryotic genomes (2540 from Bacteria and 164 from Archaea, totaling 8,422,211 proteins) (Additional file 1: Figure S1). Initially, we found that 633 gene families comprised composite eukaryotic genes with a prokaryotic origin, i.e., both the composite eukaryotic genes and at least one prokaryotic gene could be aligned over their full lengths. The origin of these composite genes likely predated LECA. We also found that composite eukaryotic genes in 383 gene families did not share detectable local similarity with prokaryotic sequences, and were thus likely to be eukaryotic innovations. Finally, 605 gene families corresponded to S-genes, because only partial sequence similarity was detected between composite eukaryotic and prokaryotic sequences. Of these 605 families, 32 were removed because of the low sequence similarity with prokaryotic sequences found by using a more sensitive procedure based on hidden Markov model (HMM) profiles. We also checked for full-length homology with genomes from the recently discovered Asgard phylum [10]. Only a single S-gene family encoding tubulin (family 403) appears to have been inherited from the Asgard group; this is not to be taken as evidence against an emergence of eukaryotes from Asgard. Rather, the limited full-length homology between eukaryotic S-proteins and Asgard proteins is compatible with the emergence of eukaryotes from the latter clade. That is, S-genes likely evolved in the branch leading to LECA, rather than in the common ancestor of LECA and its closest Asgard relative. Finally, five families were removed because the prokaryotic signal detected for the components was likely due to HGTs from eukaryotes to intracellular prokaryotes [33]. The 567 remaining S-genes are of interest because they evolved from combining and recycling at least one genetic fragment of prokaryotic ancestry, either archaeal or bacterial, usually with eukaryotic genetic fragments, within a eukaryotic host lineage.

#### The distribution of S-genes in eukaryotes identifies 282 ancient families

**Fig. 1:**
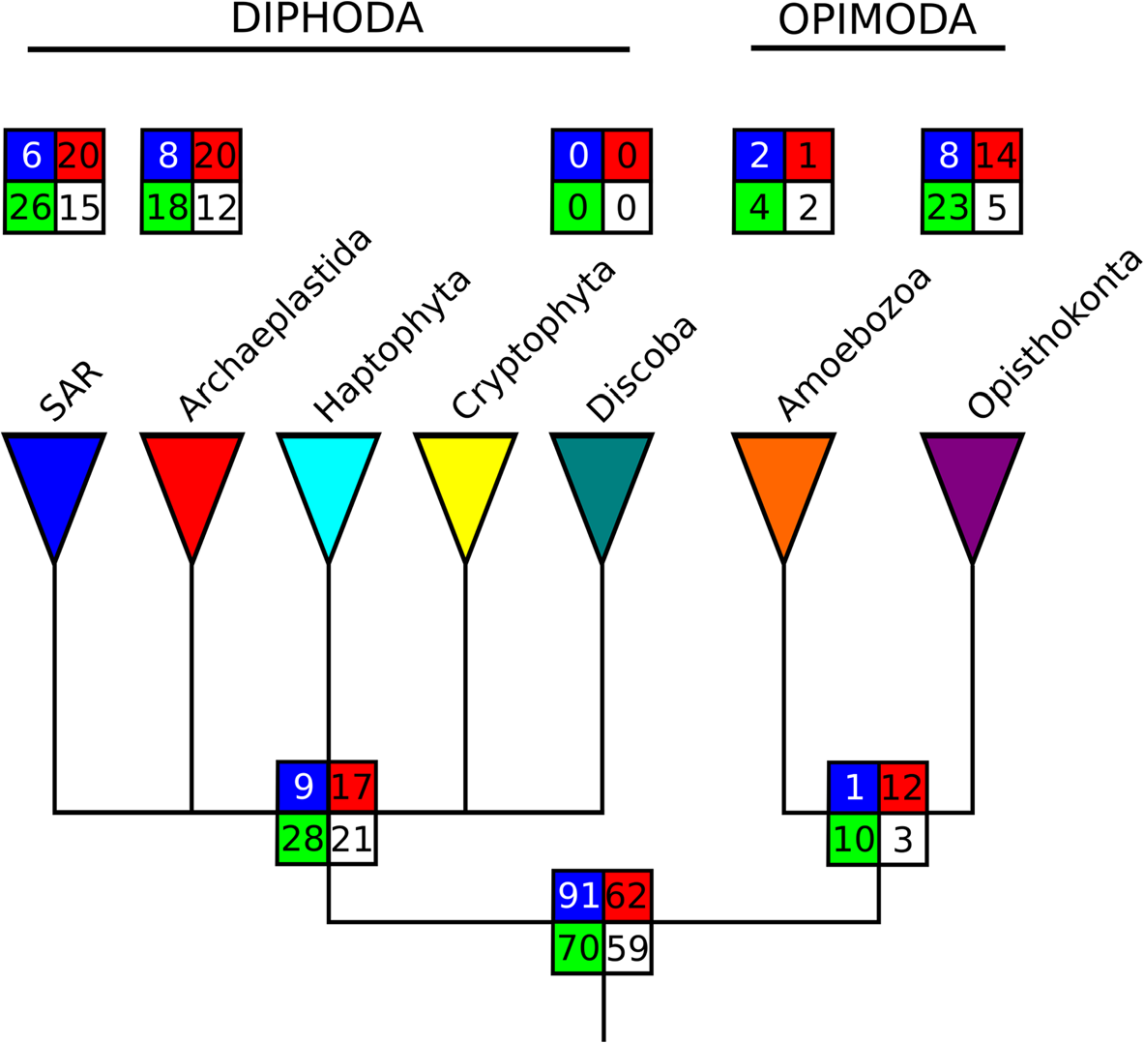
Putative phylogeny of eukaryotes, based on Derelle et al. [82], that shows the distribution of 573 S-gene families. Family evolution reconstruction was performed using Dollo parsimony. The four boxes correspond to the number of families involved in metabolism (red), information storage and processing (blue), cellular processes and signaling (green), and poorly characterized processes (white)

The distribution of S-genes across eukaryotic lineages reveals that 50% of these families (e.g., 282 gene families) are present both in Opimoda and Diphoda and therefore were likely present in LECA (Fig. 1; see also Additional file 3: Figure S3 and Additional file 4: Figure S4 for the time of emergence of S-genes based on alternative eukaryotic trees). Nonetheless, S-genes with a broad distribution across eukaryotic supergroups may be the result of two types (of mutually non-exclusive) processes. The first is that these genes may be genuinely ancient, emerging during eukaryogenesis and retained in various eukaryotic supergroups. Under this scenario, phylogenies of broadly distributed S-genes should support the monophyly of each supergroup and potentially resolve supergroup interrelationships. The second is that the broad distribution of S-genes might be the result of horizontal transfer that spread S-genes across eukaryotic supergroups. The latter includes EGT if an ancestral eukaryote engulfed another distantly related eukaryote that encoded S-genes of endosymbiont origin. Alternatively, this pattern could result from the horizontal transfer of individual S-genes between distantly related eukaryotes. In these cases, resolved individual phylogenies of the S-gene should show a mixture of sequences from different supergroups (i.e., supergroups would not be monophyletic). Distinguishing between these two processes requires well-resolved individual gene trees. To this end, we reconstructed and manually inspected maximum likelihood phylogenetic trees (Additional file 5: Sheet 1) from 255 broadly distributed S-genes (e.g., ≥ 4 sequences from S-genes with > 3 hosts, proposed to be ‘ancient’ based on their taxonomic distribution, could be aligned; see Methods). These analyses indicate that 85% (216 out of 255) of these S-genes are of ancient origin and were vertically inherited in eukaryotes. Other more complex scenarios could not be ruled out to explain the topologies of the remaining S-gene trees.

We posit that the formation of S-genes in the earliest diverging eukaryotes may be an outcome of extensive genome remodeling due to intron invasion [34] and gene duplication in the LECA [3]. We tested this hypothesis by looking at 82 anciently derived (hereafter, referred to as ‘early’) S-gene families present in six well-annotated genomes of Diphoda (*Phaeodactylum tricornutum*, *Paramecium tetraurelia*, *Chlamydomonas reinhardtii*) and Opimoda (*Dictyostelium discoideum*, *Monosiga brevicollis*, *Capsaspora owczarzaki*). We aligned the corresponding protein sequences using MAFFT [35], highlighting the position of introns, and checked manually for homologous sequences of at least one Diphoda and at least one Opimoda containing at least one intron, located at a similar position between components (i.e., ± 20 aa of a component borders). Introns shared by Diphoda and Opimoda are possibly ancient, predating the split between these two major lineages (even though convergences regarding the similar positions of these introns in different eukaryotic lineages cannot be ruled out). Among tested early S-gene families, 20 displayed at least one ancient intron between their components (Additional file 6 and Additional file 7: Figure S5), a feature consistent with the hypothesis that introns may have contributed to the evolution of some novel genes in eukaryotes [36]. Moreover, 51 S-genes families (including all 20 of the above families) presented likely ancient introns, although located within (and not between) components.

In contrast with early S-genes, S-genes with a restricted taxonomic distribution are compatible with their formation at multiple phylogenetic depths, secondary loss in multiple lineages [37], and/or gene fission [38] of ancestral S-genes. Interestingly, 32% of S-genes are present in a single eukaryotic lineage (184 families), and could serve as synapomorphies (i.e., adaptive functions) for these groups [38]. In particular, within the SAR group, ciliates contain a high proportion of exclusive S-genes (38 families, Additional file 8: Figure S6). Ciliates are known for their complex mechanisms of programmed genome rearrangements [39], which may have facilitated chimeric gene creation [40]. S-genes in ciliates do not seem to fulfil random functions, i.e., they are mostly involved in cellular processes and signaling (21 S-genes), with 13 playing a role in signal transduction mechanisms (Additional file 6).

### New essential eukaryotic components

#### Many early S-genes encode components of the informational machinery

**Fig. 2:**
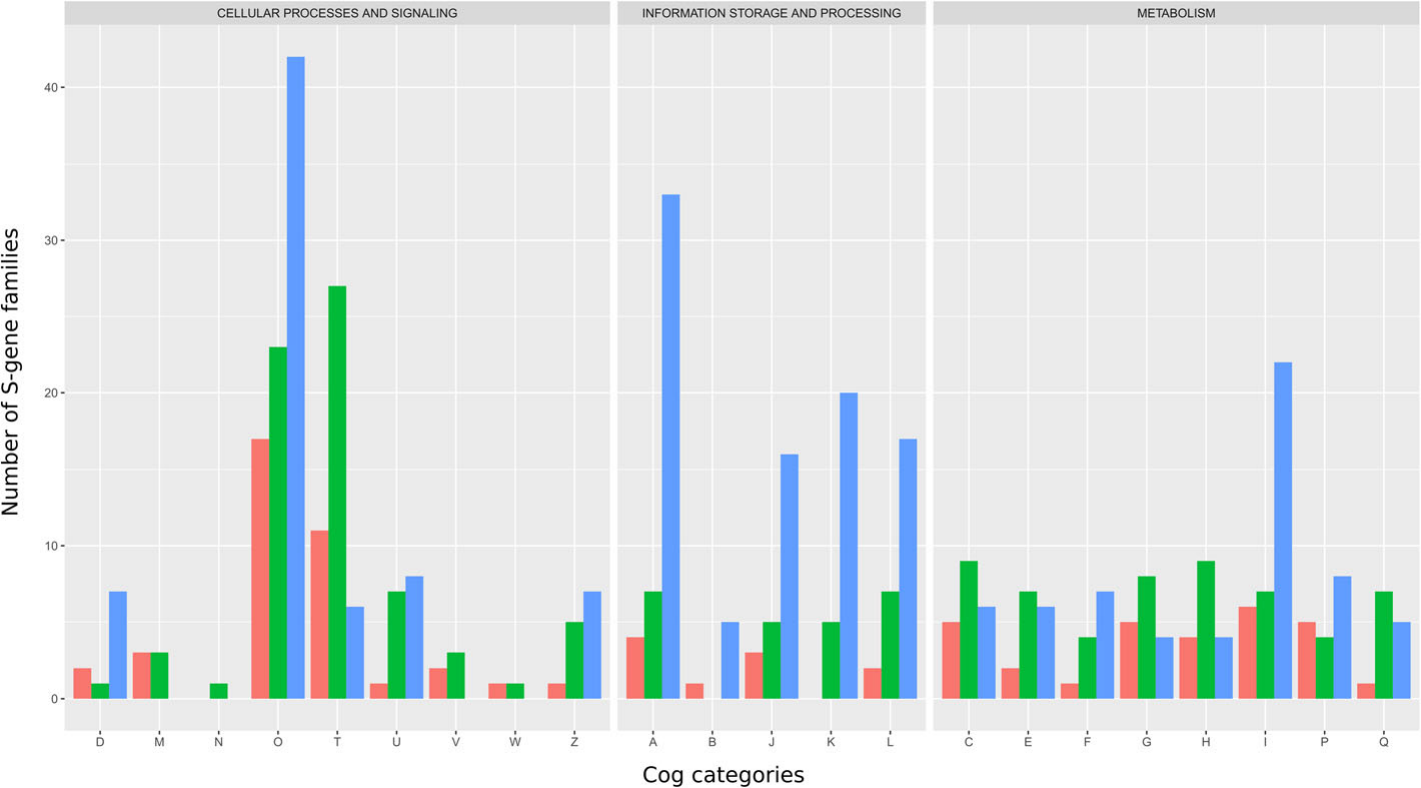
Functional annotation of the 573 S-genes based on COG categories. S-gene families were divided into early (S-genes found in both Opimoda and Diphoda, 286 gene families, in blue), intermediate (S-genes found either in Opimoda or Diphoda, 101 gene families, in pink), and lineage specific (S-genes found in one eukaryotic supergroups, 186 gene families, in green) (COG category definitions can be found here: http://eggnogdb.embl.de/download/eggnog_4.5/COG_functional_categories.txt)

Early S-genes contributed in many important ways to eukaryogenesis. Functional predictions suggest they are involved in cellular processes and signaling, primarily in the ‘O’ (Post-translational modification, protein turnover, chaperones) category, but also in the ‘U’ (Intracellular trafficking, secretion, and vesicular transport), ‘D’ (Cell cycle control and mitosis), and ‘Z’ (Cytoskeleton) categories, in information storage and processing (mainly the ‘A’ (RNA processing and modification), ‘K’ (Transcription), ‘L’ (DNA Replication and repair), and ‘J’ (Translation) categories), as well as in metabolism (particularly the ‘I’ (Lipid metabolism) category) (Fig. 2).

**Fig. 3:**
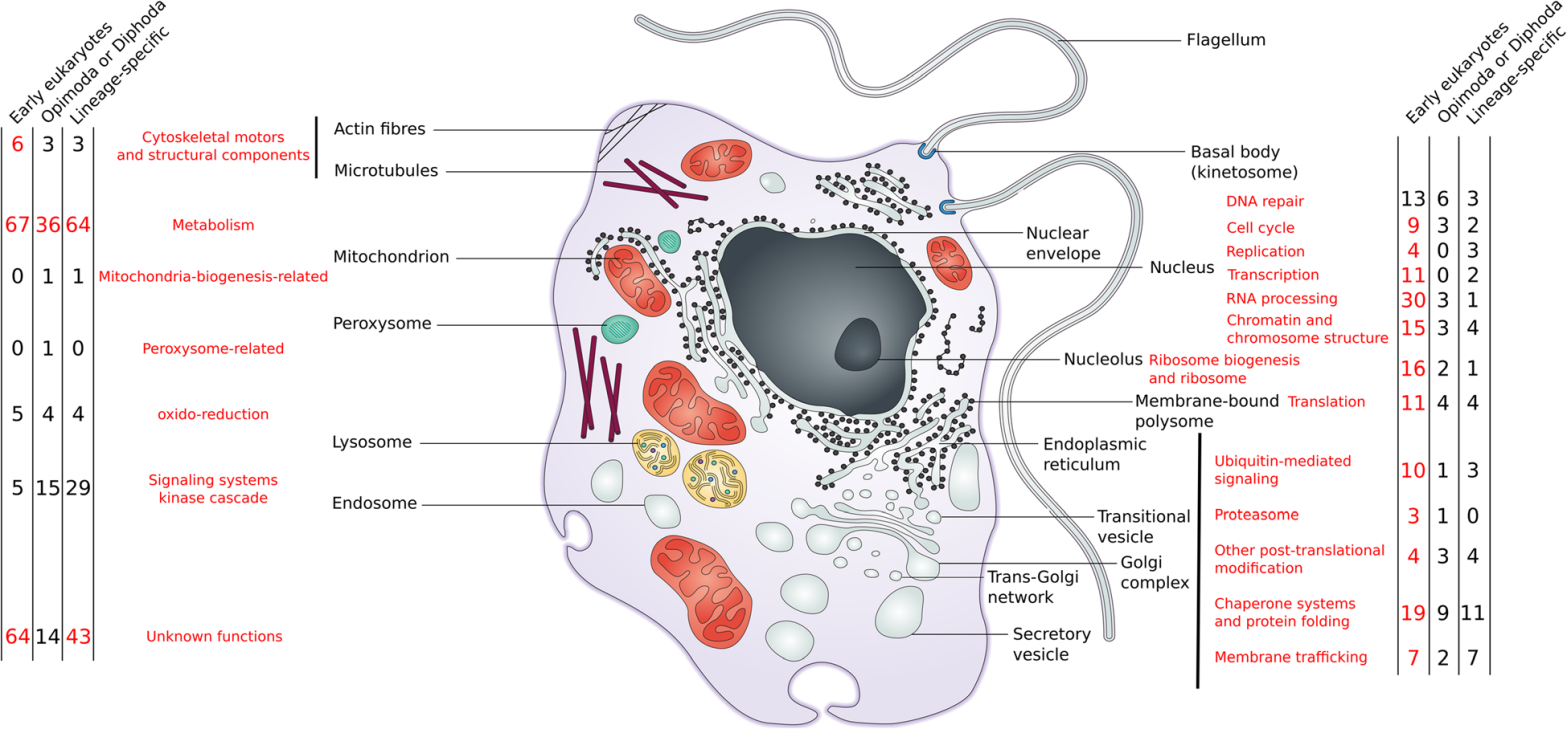
Mapping of the functions of 573 S-genes in a eukaryotic cell (figure adapted from de Duve [83]). Numbers in red correspond to functions containing essential S-genes in yeast

A detailed gene-by-gene analysis (Fig. 3, Additional file 6) substantiates the relevance of S-genes to eukaryote biology and evolution. These composite genes are key components of the replisome (families 41,894 and 8452), the spliceosome (families 5353, 14,116, and 7536), the transcriptional (families 15,440, 8572, and 31,114) and translational machineries (families 6980, 15,594, and 4775), ribosome biogenesis and assembly (families 9105, 9136, and 4331), chromatin and chromosome structure (families 3752, 5196, and 60,478), and DNA repair (families 19,268, 39,836, and 16,839) (Fig. 3, Additional file 6). S-genes augmented the informational machinery during eukaryogenesis by adding new components to existing processes [24–26]. Defense against parasitic genetic elements, such as introns, may explain why eukaryotic gene expression requires additional processing steps not observed in prokaryotes [41]. Indeed, dealing with introns was a major function of anciently derived S-genes, consistent with the notion that introns ‘plagued’ early eukaryotic genomes (Additional file 9: Figure S7). Tinkering with the DNA repair system is supported by the following observations. Prokaryotic endosymbionts within a free-living prokaryotic host have not been described thus far, indicating that this nested lifestyle is likely difficult to establish. Genotoxicity might be one of many barriers to the success of such endosymbioses [42, 43]. During early eukaryogenesis, the DNA within the proto-mitochondrion was likely adversely impacted by the chemically harsh environment resulting from the inclusion of that organelle within its host [44]. In addition, the organelle generated ROS, rendering the cellular environment toxic for host DNA if this genome was not protected by the nuclear membrane. Two out of three components of the MRX complex, involved in repairing DNA double-strand breaks using homologous recombination [45], are S-genes (families 18,347 and 18,341) that provide protection from genotoxicity. Interestingly, S-gene MRE11 (family 18,347) of the MRX complex is also involved in meiotic double-strand DNA breaks repair in *Caenorhabditis elegans* [46], suggesting a potential link between MRX S-genes and the evolution of sex. None of the yeast nuclear pore complex proteins are descended from early S-genes. This is either because LECA lacked a nucleus, implying that, in addition to a possible sensitivity to genotoxic substances, early hosts of the mitochondria presented less barriers to lateral gene transfer (LGT). Alternatively, there was a nucleus, but the nuclear pore complexes were not affected by this form of genetic remodeling (i.e., the use of a prokaryotic fragment).

#### Some S-genes may have resulted from the crystallization of ancient associations

S-gene evolution addressed yet another challenge faced by eukaryotes, namely that early eukaryotic cells were larger and more compartmentalized than individual prokaryotic cells, which presumably limited protein–protein interactions because these interactions require some form of coordinated intracellular targeting. We report 282 occurrences of the physical association of multiple domains in a single novel eukaryotic gene, whereas these domains are not so tightly connected in prokaryotes. This genetic remodeling ensured the direct interaction of these domains once translated into proteins in the eukaryotic cell. In contrast, domains encoded by separate genes are less likely to be able to interact in a larger compartmentalized cell [47]. Consistent with this notion that S-genes stabilize functional interactions, and assuming that some operons were inherited from the bacterial and archaeal partners, we infer that 19 ancestral prokaryotic operons, encoding functions such as proton transport, transmembrane transport, or DNA-templated transcription, fused into S-genes during early eukaryote evolution. The transformation of operons into S-genes facilitates the coordinated expression of interacting proteins and presumably solved the problem of decoupled transcription and translation in eukaryotes (Table 1). The sparse taxonomic distribution of 14 other prokaryotic operons suggests they evolved into S-genes later during eukaryotic evolution, or were secondarily lost from eukaryotic lineages.

**Table 1:**
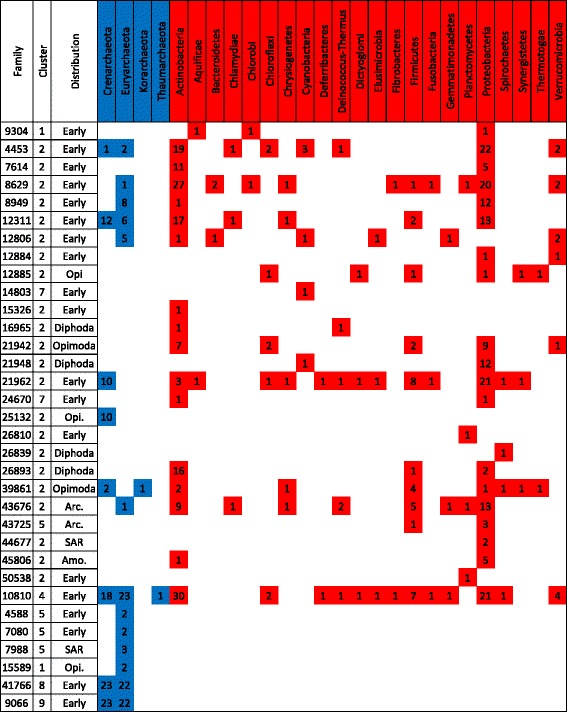
The 33 operon-like composite families, along with the prokaryotic phyla where these operons were detected

*Opi* Opisthokonta, *Arc* Archaeaplastida, *Amo* Amoebozoa

#### S-gene-encoded proteins are not enriched in targeted proteins

In silico predictions indicated that approximately 110 S-genes are targeted to organelles (19%) (Additional file 6). Among them, 34 families contain more than 50% of their members predicted as mitochondrion targeted (Additional file 6). Of note, proteins encoded by the early operon-like S-gene family 4453 are targeted to mitochondria. These genes encode the alpha and beta subunits of NAPH transhydrogenase. Another interesting S-gene is the family 3528 encoding a protein kinase (PKP2) in *Saccharomyces cerevisiae*, which negatively regulates pyruvate dehydrogenase [43]. Surprisingly, S-genes are significantly depleted in targeted proteins (Z-score −4.89, *P* = 9.93 × 10^–7^). This limited proportion of targeted S-genes contrasts with the 64% of targeted S-genes found in Kaessmann’s study [2] and can be seen as counter-intuitive. We hypothesize that this result highlights the diversity of the roles played by S-genes during eukaryogenesis. Whereas, in photosynthetic eukaryotes, the vast majority of S-genes are involved in the photosynthetic function, the challenges faced by the first eukaryotic cells extended beyond the scope of the acquisition of a novel organelle (e.g., dealing with a bigger cell and dealing with nucleic parasites).

#### Many S-genes may also have contributed to the increase of cellular complexity

Many early S-genes are involved in chaperone systems and protein folding that may also have contributed to dealing with an increase in cell complexity [3]. Six S-gene families containing a DnaJ domain and 11 S-genes with isomerase activities act as chaperones and folding catalysts (Additional file 6). S-genes are also involved in intracellular trafficking, such as the Golgi-REG interface vis-à-vis the COPI and COPII coating machineries (families 3724, 3693, 63,542, and 7977). Finally, early S-genes were frequently involved in post-translational modification and protein turnover, with at least 14 S-genes belonging to the ubiquitin system and the proteasome. These proteins, although of archaeal origin [48], are known to have diversified via architectural rearrangements in early eukaryotes with the evolution of further complexity in some lineages [49]. In a primitive eukaryotic cell already harboring complex endomembrane compartments, early developments in post-translational and trafficking systems were likely to have been advantageous. Early S-genes also contributed metabolic functionality with involvement in lipid transport and metabolism, with six represented in glycerophospholipid metabolism, which is important for membrane biogenesis (Additional file 10: Figure S8). Of note, subsequent lineage-specific tinkering of metabolic S-genes was an important process as illustrated by the number of metabolic S-genes with a lineage-specific distribution (Additional file 8: Figure S6).

Overall, the 567 S-genes detected in this analysis (with 282 presumably present in the LECA) contributed to important cellular systems and processes in eukaryotes (Figs. 2 and 3). In the model organism *S. cerevisiae*, 44 out of 113 existing S-gene families are essential (Additional file 6) (a higher ratio when compared to the ratio of essential genes [103] in non-symbiogenetic composite gene families [341]). S-genes also have a higher degree in the yeast PPI networks (median = 36.00; 1 sr Qu. = 18.50; 3rd Qu. = 56.00) than other composite genes (median = 26.00; 1 sr Qu. = 14.00; 3rd Qu. = 45.00), indicating they associate with a higher number of protein partners (Additional file 6). This essentiality and high degree in PPI networks of S-genes is explicable because 51 of them encode proteins involved in macromolecular complexes, 34 of which contribute to key eukaryotic informational macromolecular machineries in yeast (Additional file 6).

### Phylogenetic origins of S-genes

#### Taxonomic assignment of the components of S-genes

**Fig. 4:**
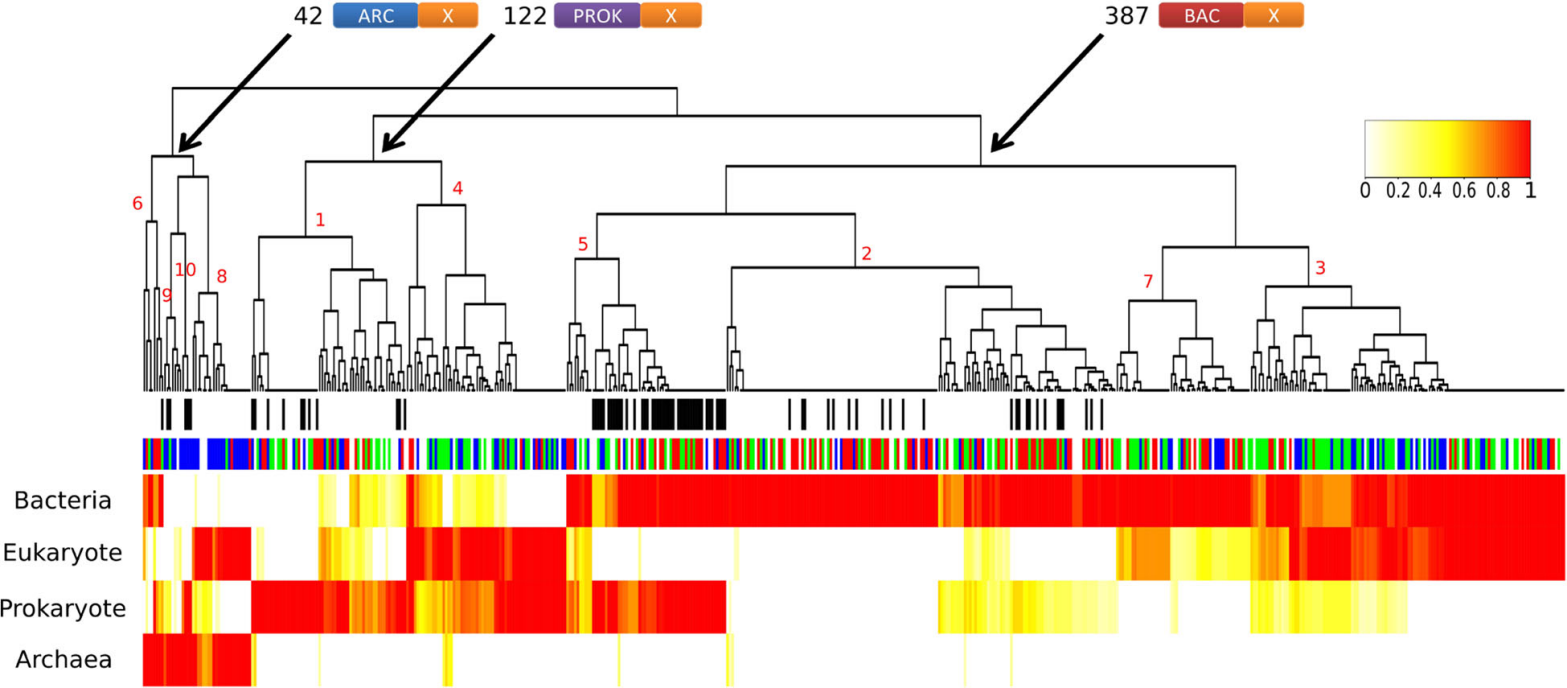
Hierarchical clustering of S-gene families according to their component origins. The heatmap represents the ratio of genes in a given family (columns) that have at least one component of a given origin (eukaryotic, archaeal, bacterial or prokaryotic; the rows). White lines correspond to the absence of a component from a given origin in every gene in the given S-gene family. The colored lines correspond to the presence of at least one component of the given origin in a given percentage of genes in the given S-gene family (red lines denote that all (100%) genes contain a given origin component). The first colored top bar indicates the functional annotation. The black bars in the second colored top bar indicate the reclassified S-genes after applying the HMM-profile procedure. Cluster 1 roughly corresponds to 60 S-genes with only prokaryotic components (PROK-PROK), clusters 2 and 7 roughly correspond to 203 S-genes with only bacterial components (BAC-BAC), cluster 3 roughly corresponds to 122 S-genes with bacterial and eukaryotic components (BAC-EUK), cluster 4 roughly corresponds to 62 S-genes with prokaryotic and eukaryotic components (PROK-EUK), cluster 5 roughly corresponds to 62 S-genes with prokaryotic and bacterial components (PROK-BAC), cluster 6 roughly corresponds to 8 S-genes with bacterial and archaeal components (ARC-BAC), cluster 8 roughly corresponds to 23 S-genes with archaeal and eukaryotic components (ARC-EUK), cluster 9 roughly corresponds to 7 S-genes with only archaeal components (ARC-ARC), and cluster 10 roughly corresponds to 4 S-genes with prokaryotic and archaeal components (ARC-PROK)

The origin of each S-gene component (i.e., archaeal, bacterial, or prokaryotic in general) was identified based on the top ten BLASTP hits (see Methods and Additional file 11: Figure S9). When components were only found in eukaryotes (lacking a match with any prokaryotic sequence), we performed a HMMER search to confirm that the components of these S-genes were not homologous to prokaryotic sequences that may have diverged beyond recognition using BLASTP (see Methods and Fig. 4). A straightforward interpretation for components limited to eukaryotes is that they evolved after eukaryogenesis and have a non-symbiogenetic origin as eukaryote-specific components.

We also performed maximum likelihood phylogenetic analyses of the 429 S-genes with at least one archaeal (Additional file 5: Sheet 2) or bacterial (Additional file 5: Sheet 3) component to confirm our BLAST-based assignation of these components. All of these phylogenies were both bioinformatically and manually inspected to assign an origin to each component of the 429 S-genes (e.g., archaeal or bacterial, or when uncertain regarding the prokaryotic domain of its origin, simply prokaryotic). We used several criteria to interpret these trees. First, although we looked at the 500 top hits, some component trees were exclusively comprised of (1) either archaeal and eukaryotic sequences, or (2) bacterial and eukaryotic sequences. For those trees, the origin of the components is clear, in agreement with the BLAST assignation. Second, we rooted the component trees harboring bacterial, archaeal, and eukaryotic sequences between Bacteria and Archaea, when possible (i.e., when ancient paralogy and LGT between Archaea and Bacteria did not prevent such a conventional rooting of the component tree). In this set of rooted phylogenies, we tested whether the eukaryotic components from the S-genes were nested in the Archaea (or in the Bacteria), and were monophyletic. This approach allowed us to confirm the archaeal or bacterial origin of the components of S-genes (Additional file 5: Sheets 2 and 3). Finally, the remaining trees were inspected manually. Regarding 410 out of the 429 families for which phylogenetic trees of bacterial and archaeal components could be reconstructed, 320 families returned phylogenetic trees of components that are consistent with the BLASTP assignment, 15 families show inconsistent phylogenetic trees and BLASTP assignations, 39 families have inconclusive (i.e., too weakly resolved) phylogenetic trees for all their components, and 36 families are only inconclusive for some of their components, i.e., they have at least one inconclusive phylogenetic tree for a component, yet at least another informative phylogenetic tree, for a different component, that is consistent with the origin assignment based on BLASTP. These two independent analyses agree for the majority, and most importantly, are largely not incongruent (only 15 families showing inconsistency). They also identify 320 S-gene families that are supported both by the phylogenetic analysis and the BLASTP assignation, whereas 90 S-gene families are assigned to a given phylogenetic origin only based on the BLASTP inference (Additional file 6). A finer-grained analysis of the origins of the bacterial components identified two bacterial phyla, the Proteobacteria (26%) and the Cyanobacteria (21%), as major contributors (Additional file 12: Sheet 2). This is compatible with the notion that the ancestors of the mitochondria and plastids are the most important contributors to bacterial genes in eukaryotes [37]. However, additional phyla contributed to S-genes, including Firmicutes (9%), Chloroflexi (6%), Bacteroidetes (6%), and Actinobacteria (6%), indicating multiple bacterial donors to the eukaryotic gene inventory, as proposed by Pittis et al. [18] (but see [50, 51] for alternative explanations for this apparent diversity of bacterial sources).

#### Components of S-genes do not associate randomly

Clustering S-genes based on the phylogenetic origin of their components showed that components do not associate randomly (Fig. 4). Very few S-genes (only 8; cluster 6 in Fig. 4) have combined fragments of archaeal and bacterial origins. This result might be surprising if one considers that genetic fragments from these two prokaryotic sources have co-occurred in the same genome for about two billion years [11]. In fact, most S-genes (387; clusters 2, 3, 5, and 7) contain a component of bacterial origin that is either combined with another bacterial (203; clusters 2 and 7) or eukaryotic component (122; cluster 3), whereas only 42 S-genes with a component clearly of archaeal origin were identified (clusters 6, 8, 9, and 10). In order to understand this limited number of S-genes derived from Archaea, we looked in detail at clusters 1 (60 S-genes), 4 (62 S-genes), 5 (62 S-genes), and 10 (4 S-genes), which correspond to S-genes with components of prokaryotic origin (i.e., components similar to prokaryotes that we cannot assign to Archaea or Bacteria, according to our parameters). We observed that 47, 37, 35, and 2 families in clusters 1, 4, 5, and 10, respectively, contain at least one archaeal sequence in the top three hits of their components (Additional file 12: Sheet 1). These observations suggest that some families in these clusters may contain components of archaeal origin that are identified as prokaryotic because of the limited number of genomes available from Archaea.

We also looked in detail at the phylogenetic trees of the 42 S-genes with at least one archaeal component to verify that our approach did not miss the ‘ultimate’ origin of some of these archaeal components. This addresses the possibility that these sequences originated from bacterial genomes, and were then transferred to an archaeal major group (consistent with previous work [52, 53]), before being inherited by eukaryotes. If the eukaryotic components for which BLAST assigned an archaeal origin were nested within Archaea in a rooted tree of life, then the ‘proximate’ origin of such bacterial-then-archaeal-components would still be archaeal, because they entered eukaryotes via the archaeal partner. To determine whether the ultimate origin of the eukaryotic component might nonetheless be ‘bacterial’, we analyzed the taxonomic distribution within the archaeal (and eukaryotic) clade on the one hand, and the taxonomic distribution within the bacterial clade on the other. We reasoned that, if the component gene/domain present in the Archaea was acquired from Bacteria at the time one major archaeal group evolved, then the diversity of Archaea hosting this gene/domain should be restricted to one archaeal major group. When that was the case, the tree topology could suggest that the gene was first transferred from a bacterium to an archaeum, then inherited from an archaeum, and subsequently recycled and used as a component in a eukaryotic S-gene. We identified two such components (Additional file 5: Sheet 2). When, by contrast, the taxonomic distribution of Archaea was broader than a single archaeal major group and, likewise, when the taxonomic distribution of Bacteria was also broad, the component gene/domain was likely of ancient prokaryotic origin (i.e., originated before the split of Archaea and Bacteria), and the component tree provided no positive evidence for an ultimate bacterial origin; there were 19 such components. Finally, a third class of 25 component trees required visual inspection (because of ancient paralogy, recent LGTs, or phylogenetic artefacts leading to complex relationships between taxa; Additional file 5). In those trees, only two appeared compatible with an ultimate bacterial origin. Thus, the results showed that S-genes are largely of bacterial origin, whereas S-genes with archaeal components are more rare, which is consistent with the analysis of full-length genes [54].

**Fig. 5:**
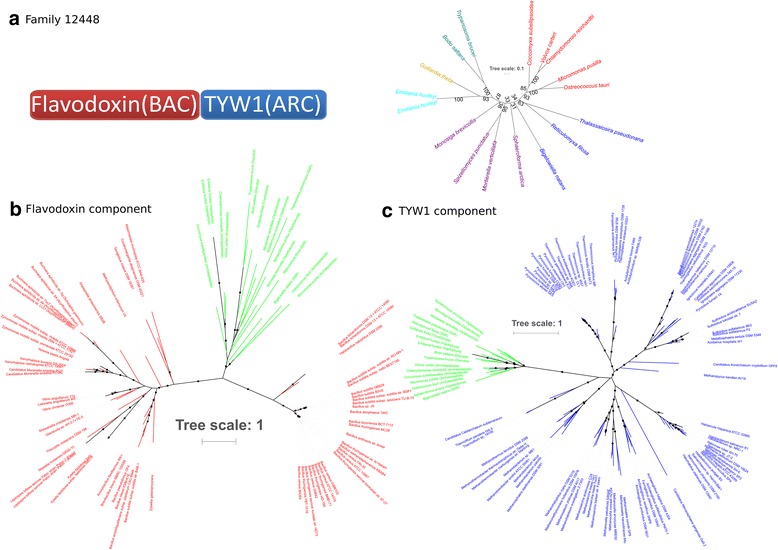
S-gene family 12,448. **a** Component architecture and phylogenetic tree of S-gene family 12,448. Family 12,448 is composed of two components (Flavodoxin and TYW1) of bacterial and archaeal origins, respectively, according to our BLASTp taxonomic assignment (for the phylogenetic tree, blue: SAR, red: Archaeplastida, purple: Opisthokonta, cyan: Haptophyta, yellow: Cryptophyta, blue-green: Discoba) (17 sequences, 407 sites, model LG + G4, 1000 ultrafast bootstraps). **b** Maximum likelihood (ML) phylogenetic tree of the flavodoxin component (green: Eukarya, blue: Archaea, red: Bacteria, black circle: bootstraps > 80%) (117 sequences, 164 sites, model LG + G4, 1000 ultrafast bootstraps). **c** ML phylogenetic tree of the TYW1 component (green: Eukarya, blue: Archaea, red: Bacteria, black circle: bootstraps > 80%) (117 sequences, 246 sites, model LG + I + G4, 1000 ultrafast bootstraps)

Specific examples of these S-gene categories will help highlight the diversity of their origins during eukaryogenesis. For example, family 12,448 (Fig. 5a) illustrates the merging of components from very different origins. This ARC-BAC S-gene family is involved in the biosynthesis of the hypermodified tRNA base wybutosine [55], which enhances the accuracy of translation [56]. Although in eukaryotes, the wybutosine biosynthesis pathway is likely derived from the archaeal ancestor [57], we report here the fusion of a bacterial domain. This results in a protein with a unique domain architecture consisting of an N-terminal flavodoxin region of bacterial origin (Fig. 5b) and a C-terminal catalytic domain TYW1 of archaeal origin [57] (Fig. 5c). TYW1 is a member of the radical SAM superfamily that binds iron-sulfur clusters. The role of the flavodoxin-like domain is not known; however, all radical SAM enzymes require the reductive activation of the iron-sulfur cluster by an external reductant which, in vivo, is thought to be flavodoxin or a related protein [55]. Thus, this association of two domains could have resulted in an emergent property at the level of S-proteins, i.e., the bacterial domain reduced the iron sulfur cluster of the archaeal domain, without the need for an external reductant.

Family 18,563 illustrates a more common combination of components. This ARC-EUK S-gene family encodes proteins with three domains (Additional file 13: Figure S10A) that associate an RNA methyltransferase of archaeal origin (Additional file 13: Figure S10B) with two domains that lack hits to prokaryotes (and thus are of eukaryotic origin). In yeast, this S-protein, named Spb1p, is nucleolar and essential [58]. Spb1p is required for ribosome synthesis [58] because it catalyzes the methylation of guanine at position 2922, a universally conserved position at the catalytic center of the ribosome that is essential for translation, during maturation of the 27S pre-rRNA [59].

An even more common type of domain association involves components with a bacterial origin with components of eukaryotic origin. This type of fusion is exemplified by the evolution of a subunit of a translation elongation factor, family 6384 (Additional file 14: Figure S11A). Eukaryotic translation elongation factors (eEF) include eEF1A and eEF1B, which recruit aminoacyl-tRNAs onto the ribosome [60]. S-gene family 6384 encodes the gamma subunit of translational elongation factor eEF1B. These S-proteins are comprised of two domains, which are connected through a highly polar central lysine-rich stretch of residues (Additional file 14: Figure S11A). The N-terminal region encodes a glutathione S-transferase domain of bacterial origin [61] (Additional file 14: Figure S11B), whereas, although highly conserved in eukaryotes, no sequence or structural homology with known functional domains has thus far been described for the C-terminal region [60]. This region of eukaryotic provenance has been proposed to interact with another eEF1B gamma subunit to support the quaternary structure of the eEF1B complex [60]. In humans, the N-terminal region interacts with the alpha and epsilon subunits [62]. No clear enzymatic function has been associated with eEF1B gamma proteins, but it is likely that its main role is to ensure the proper scaffolding of the different subunits in the eEF1B complex, as well as to direct its intracellular localization [60]. The eEF1B gamma subunit is also a substrate for CDK1/cyclin B, suggesting its possible role in the control of expression during the cell cycle.

Finally, some S-genes reinforce pre-existing interactions between proteins (and their domains). This class of S-genes is illustrated by family 9304, characterized by Gawryluk et al. [63]. This family associates components of prokaryotic origin (Additional file 15: Figure S12A) that are organized in an operon. In eukaryotes, the S-gene family encodes an ATP-citrate lyase of two distinct and fused subunits A and B (Additional file 15: Figure S12B and S12C). ATP-citrate lyase catalyzes the ATP-dependent cleavage of citrate into oxaloacetate and acetyl-CoA, a key metabolite because acetyl-CoA is involved in multiple essential metabolic pathways in eukaryotes [64]. Interestingly, the phylogenetic trees corresponding to each subunit are congruent, strongly suggesting that the two subunits have a shared history (Additional file 15: Figure S12B and S12C), consistent with the existence of a selective pressure for their co-occurrence in genomes.

#### Functions of S-genes correlate with their component origins

The phylogenetic origin of S-gene components also correlates with functions (Fig. 4). S-genes with archaeal components (clusters 6, 8, 9, and 10 in Fig. 4) (42 S-genes) are primarily associated with informational functions (28/42) (χ^2^ test, adjusted *P* = 0.00311, Additional file 15: Figure S12), whereas S-genes of bacterial origins (clusters 2, 3, 5, and 7 in Fig. 4) are primarily involved in operational functions, typically metabolism (clusters 2 and 7, 79/203 S-genes involved in metabolism) (χ^2^ test, adjusted *P* = 0.03094, Additional file 16: Figure S13). S-genes with bacterial and eukaryotic components are enriched in cellular processes and signaling such as signal transduction (13 families), chaperones (8 families carry DnaJ domain), or trafficking (10 families) (54/122 S-genes in cluster 3 are involved in cellular processes and signaling) (χ^2^ test, adjusted *P* = 0.07106, Additional file 16: Figure S13). At first glance, the evolution of S-genes thus seems consistent with the findings by Rivera and Lake [19] on the origin of eukaryotic genes, i.e., intact genes inherited from an archaeal ancestor are involved in informational functions, whereas intact genes of bacterial origin are involved in operational functions. However, although this correlation exists for S-genes in relative proportion, when the number of families is considered, S-gene families with bacterial origins encode twice as many informational processes (62) than S-gene families with archaeal origins (28). In yeast, for 209 described informational genes [54], a vast majority are of archaeal origin (146). However, for the subset of these informational genes that we classified as S-genes, the proportions are shifted. Thus, even though S-genes only represent about 19% of the yeast informational genes, their evolution contrasts with that of informational genes in general, and is strongly impacted by genes of bacterial origin.

Thus, there is a large hidden bacterial contribution to the evolution of eukaryotes, beyond operational functions, consistent with the work of Cotton and McInerney [21]. Identifying a dominant bacterial signal in eukaryotic informational genes may be explained by the inability to identify bona fide archaeal homologues due to a much smaller database of available genomes from these prokaryotes. Regardless, we feel that explaining this finding is not trivial, and we can only speculate. Because the host was likely an archaeum, replacing genes that encode a significant fraction of the informational machinery of archaeal origin with bacterial genes might have been counter-selected in chimeric eukaryotic lineages. However, modifying minor components of this machinery may have been less detrimental given the ‘rain’ of bacterial DNA originating from the mitochondrial endosymbiont, or possibly from other bacterial symbionts [18, 65]. Another explanation for the seemingly higher evolvability of bacterial genes may come from a more specific consideration of the informational processes, i.e., these S-genes with bacterial domains are largely involved in RNA processing (Additional file 17: Figure S14). For example, 10 such S-genes are associated with the spliceosomal machinery (Additional file 9: Figure S7), and therefore these informational bacterial components may theoretically derive from the genome of the mitochondrial ancestor. Indeed, the spliceosome, a new informational machinery that evolved in eukaryotes, appears in part to be derived from group II introns – thus from bacterial DNA [66]. However, analyses of trees from the uncontroversial bacterial components of these S-genes did not recover a signal for such an Alpha-proteobacterial origin. The complementary observation, a possible subgenic contribution of Archaea to eukaryogenesis, is not supported by our data. That is, Archaea did not contribute many genetic fragments to S-genes associated with operational genes in eukaryotes. Thus, not only at the gene level [54], but also at the subgenic level, the evolvability of genes derived from Archaea appears more limited than that of Bacteria in nuclear genomes. Whereas S-genes with bacterial components are found in all functional categories, this is not the case for S-genes with archaeal components.

#### Small domains do not impact the results

The results described above were further critically assessed to account for the fact that assigning a phylogenetic origin to small/low complexity domains is challenging. Importantly, many families of S-proteins (174 out of 567, 30%) carry such small domains (e.g., DnaJ, zinc finger, EF-hand) and/or domains of low complexity (e.g., WD40, Leucine Rich Repeat, Ankyrin repeat) (see Methods for the full list of domains). These domains are frequently re-used in multidomain proteins [67] and tend to be involved in physical interactions. They have been linked with the evolution of eukaryotes and of organismal complexity [67, 68]. For these reasons, it is important to note that 30% of S-genes are comprised of such small and low complexity domains. Among the domains present in S-proteins, WD40 is the most frequent (64 S-protein families). In *S. cerevisiae*, 16 S-gene families contain WD40 domains, all of which are ancient and 10 of which have been found to be essential (Additional file 6). WD40 acts as a scaffold to recruit other molecules [69, 70], consistent with the finding that 9 S-proteins are involved in macromolecular machines such as the processome and the spliceosome (Additional file 6). Of note, three families are annotated only as WD40 domains (families 3840, 6543, 9846); however, two of them have known functions in ribosome biogenesis (PWP1, family 9846) [71] and in the spliceosome (CDC40/PRP17, family 6543) [72], confirming that, in spite of their simple domain architectures, these proteins have important roles in the cell. Regardless of their biological importance, the phylogenetic origin of WD40 domains is a matter of discussion because the results pinpoint to a cyanobacterial origin (Additional file 12), although many of the families carrying WD40 domains, such as COPI (family 3693), COPII (family 3724), PWP1 (family 9846), and PWP2 (family 5265), are pan-eukaryotic, which is inconsistent with the current knowledge about eukaryogenesis. This can be due to bad taxonomic assignment. We verified that our initial assignments of the origins of small and low complexity domains to archaeal or bacterial origins did not bias our results or explain the trends with regard to phylogenetic origins and functions of S-genes. To this end, we re-analyzed the data under a more conservative assumption, specifying that all small/low complexity domains are generally assigned to a prokaryotic origin, i.e., not specifically bacterial or archaeal (Additional file 6). This approach resulted in the same conclusions concerning S-genes, indicating that the presence of small and low complexity S-gene domains did not strongly impact our inferences (Additional file 18: Figure S15 and Additional file 19: Figure S16).

## Conclusions

Given the complex nature of eukaryogenesis, it is not surprising that valuable genetic information was exploited in many different ways to remodel host cell biology. Our results demonstrate that S-genes were a key part of this process, with 282 composite sequences having formed during the early phases of eukaryogenesis. We propose that these S-gene families helped address many of the challenges faced by early eukaryotes by enhancing the informational machinery, processing spliceosomal introns, countering genotoxicity within the cell, and ensuring functional protein interactions in a larger, more compartmentalized cellular environment. Moreover, it is surprising that only 42 S-genes contain an archaeal domain, which, on a per-gene basis, is about nine-fold less than that provided by Bacteria. Furthermore, in terms of the absolute number of gene families, Bacteria made a two-fold greater contribution to informational functions than Archaea. Therefore, fundamental eukaryotic properties do not strictly follow the traditional informational/operational divide for archaeal/bacterial contributions to eukaryogenesis.

## Methods

### Dataset construction

A protein sequence database was assembled by downloading every archaeal, viral, and plasmid genome that was annotated as ‘complete’ according to the NCBI Genome database on November 2013 (152, 3769, and 4294 genomes, respectively). Regarding Bacteria, one representative genome was chosen randomly per eubacterial family (230 genomes). Finally, 38 unicellular eukaryotic genomes and their organelle genomes were sampled across the eukaryotic tree of life – 19 for photosynthetic organisms and 19 that are non-photosynthetic, with a comparable total gene number and phylogenetic diversity in their ribosomal proteins. The resulting 2,192,940 protein sequences were used to perform an all-versus-all sequence comparison using BLASTP [30] (version 2.2.26) (30% protein identities cut-off in agreement with [73], *E*-value cutoff 1 × 10^–5^ and using the soft-masking parameter for low complexity regions) (see Additional file 20 for the list of genomes used).

### Domain and functional annotations

Domains were predicted using the Conserved Domain Database [31] (CDD) (version 3.13) (default parameters) and Pfam [32] (version 29.0) (default parameters). Sequences were functionally annotated by the category of their best HmmScan [74] match (version 3.1) (*E*-value cutoff 1 × 10^–5^) against eukaryotic EggNog database [75] (version 4.5). *S. cerevisiae* genes were annotated with the DEG database [76] (version 13.3) and protein–protein interactions with the BioGRID database [77] (version 3.4.136).

### Detection of S-gene families

Composite proteins were detected using FusedTriplets [29] (*E*-value < 1 × 10^–5^) by scanning the BLASTP output. All sequences were also independently clustered into protein families according to published methods [1]. Briefly, an undirected graph was constructed in which each node corresponds to a sequence and two nodes are linked if the corresponding sequences show a BLAST hit with an *E*-value < 1 × 10^–5^, ≤ 30% sequence identity, and a mutual sequence overlap of ≥ 80%. Connected components in this graph were considered protein families. Families with only eukaryotic sequences, at least three different eukaryote species, more than 50% of genes detected as composite by FusedTriplets, and with at least two domains, were kept for further analysis. In order to verify that no proteins from families have full-length homologs with prokaryotic sequences, each protein was blasted against an extensive prokaryotic dataset including Asgard genomes (2540 from Bacteria and 164 from Archaea, 8,422,211 proteins). If all sequences of a family lacked full-length homologs (i.e., no mutual alignment coverage > 80%) but showed partial similarity with prokaryote sequences, the composite family was considered an S-gene family. These families were used to create HMM-profiles using hmmbuild (default parameters) from the HMMER [74] suite (version 3.1b2) to search for distant homology. The corresponding HMM-profiles were used to screen the extended prokaryotic dataset using hmmsearch (*E*-value: 1 × 10^–5^). When a full-length match (≥ 80% mutual coverage) was identified between an S-gene and a prokaryotic gene, the corresponding family was removed from the list of S-gene candidates.

In theory, one S-gene could ultimately encode a non-S-protein, if the prokaryotic domain(s) of this S-gene were spliced out. However, since we analyzed S-proteins here, by definition, the pools of S-proteins we detected had to be associated with S-genes (i.e., composite genes including some prokaryotic domains). Therefore, in this work, we used the terms S-genes and S-proteins interchangeably. For each S-gene, prokaryotic component sequences were clustered into component families according to the following rule: if two component sequences overlapped by more than 70% of their lengths on the protein composite, they belonged to the same component family. A refining procedure was performed to merge overlapping and/or nested component families. Two component families were merged if one family was included by more than 70% of its length into the other.

Within each S-gene family, each component sequence received a taxonomic annotation by BLAST, based on the top 10 BLAST hits with prokaryotic sequences. Namely, if the ten best prokaryotic BLAST hits for a component sequence, according to the BLASTP bitscore against the composite gene, matched with a unique prokaryotic domain (e.g., Archaea or Bacteria), the component was considered to have originated from that prokaryotic domain. If there were less than ten best prokaryotic BLAST hits for a component sequence, or the ten best BLAST hits returned both archaeal and bacterial sequences, the component was considered to originate from prokaryotes. This assignation was realized for all individual components within an S-gene family, and subsequently summarized to represent the proposed origin(s) of homologous components within that S-gene family (Additional file 10: Figure S8). Thus, each S-gene family received a ratio that represented the proportion of the components with a given origin (ARC, BAC, PROK, EUK).

A more conservative taxonomic assignment was performed by considering the components carrying the following small and low complexity domains (according to the CDD database) as prokaryotic (LRR_4, WD40, LRR_RI, ANK, Kelch_1, Kelch_2, Kelch_3, Kelch_4, Kelch_5, Kelch_6, TPR, TPR_1, TPR_2, PPR, UBQ, MORN, FNI, Ube1_repeat1, Ubiquitin, TF_Zn_Ribbon, Zn-ribbon_TFIIS, UBL, RCC1, Ube1_repeat2, EFh, Kelch, FNIP, TPR_10, TPR_17, Zpr1, zf-ZPR1, UBA_EF-Ts, Ubox, S1, ZnF_C3H1, DnaJ, RING, UBA_PLICs, UBL, ZnF_C3H1).

The presence of components exclusively found in eukaryotes (i.e., without prokaryotic sequence hits) does not demonstrate that these domains are truly of eukaryotic origin. We cannot exclude the possibility that high divergence in eukaryotes and/or prokaryotes decreased sequence similarity to prokaryotic domains beyond recognition by BLAST. We tried to minimize the number of such potential false positives by using HMMER to detect distant homology. All components with proposed eukaryotic origins were used to build HMM-profiles with hmmbuild (default parameters). These profiles were used to screen the prokaryotic sequences from the extended prokaryotic dataset using hmmsearch (*E*-value: 1 × 10^–5^). All of these results were manually inspected.

### Operon-like composite detection

Operon-like composites were detected using the ProOpDB database [78], in which 191/382 genomes used in this study are referenced. Briefly, if two components of a composite were found in an operon in the same prokaryote, the composite was considered as an operon-like composite.

### Subcellular localization and enrichment test

Subcellular localizations were predicted using TargetP [79] (version 1.1b) using –P parameters for genes carried by photosynthetic organisms and –N for others. If more than 50% of the members of a family were predicted as targeted to a particular location, then this was taken to represent the family. In order to test if these S-proteins are enriched in targeted proteins, 573 non-S-protein families were randomly sampled 100 times.

### Phylogenetic analysis

Protein sequences were aligned using MAFFT [35] (version 7.222) (parameter: -linsi). Multiple sequence alignments were trimmed using trimAl [80] (version 1.4.rev15) (parameter: -automated1). Phylogenetic trees were inferred using the maximum likelihood method implemented in IQ-TREE [81] (version 1.4.4) (parameters: --TEST -bb 1000). For each reconstruction, the best model was selected using the --TEST parameter and 1000 ultrafast-bootstraps were computed.

Regarding the phylogenetic reconstructions of the full-length gene families, the relative age of broadly distributed S-genes was determined as follows. For each S-gene tree, we retrieved all its resolved partitions (with bootstrap support ≥ 85%) and verified that these partitions were not mixtures of sequences from eukaryotic supergroups. A total of 147 phylogenetic trees (e.g., 58% of the trees) did not show such mixing of sequences from different supergroups. Therefore, for all these S-genes, there is a priori no positive evidence of introgression of S-genes and the broad distribution of these S-genes is likely explained by vertical descent since they evolved in the LECA. A total of 108 phylogenetic trees showed at least one resolved partition with sequences from different supergroups. These 108 trees were visually inspected and conservatively interpreted. First, we verified whether Opimoda and Diphoda were mixed in this/these partition(s). When sequences from these two groups are not mixed, then there is no positive evidence for an LGT between these major groups, and therefore the distribution in Opimoda and in Diphoda is likely to be explained by ancient vertical acquisition in their last common ancestor. Second, we verified whether only Diphoda were mixed in this/these partition(s), which is compatible (and even expected) in case of EGT and LGT or contamination. Based on these observations, we assigned a putative age to the gene family as old, if the S-gene distribution is primarily explained by vertical descent; likely old, when the mix between Opimoda and Diphoda was limited (for example, compatible with recent LGT that might explain the presence of the s-gene in some taxa but not in all of these taxa); and inconclusive, when LGT between Opimoda and Diphoda could not be ruled out.

Regarding the phylogenetic reconstructions of the components of S-genes, HMM profiles of components were built and used to screen the prokaryotic database. For each profile, the 500 prokaryotic sequences having the best bitscores were retrieved (hmmsearch *E*-value: 1 × 10^–5^). When possible, the top 50 archaeal and 50 bacterial sequences were kept for the phylogenetic reconstructions. The archaeal or bacterial origins of components were determined as follows. First, component trees were exclusively comprised either of archaeal and eukaryotic sequences, or of bacterial and eukaryotic sequences. For those trees, the origin of the component is trivially archaeal (or bacterial). Second, for trees harboring bacterial, archaeal, and eukaryotic sequences, trees were rooted between bacteria and archaea, when possible (i.e., ancient paralogy and LGT between archaea and bacteria may prevent such a conventional rooting of the component tree). Next, in this set of rooted trees, we tested whether eukaryotic components from the S-genes were nested in the Archaea (or in the Bacteria). Third, the remaining trees were manually inspected. When the phylogeny does not allow the origin assignment, the trees were considered as inconclusive.

## Additional files

Additional file 1: **Figure S1.** Protocol used for the detection of S-gene families. A. Sequences have been clustered in gene families. B. Composite genes have been detected using FusedTriplets. C. Gene families detected as composite and having at least two domains have been kept for further analysis. D. Composite gene families only found in eukaryotes and having at least one component of prokaryotic origin were considered as S-gene families. (PNG 970 kb)

Additional file 2: **Figure S2.** Two-dimensional density graph of percentage of families detected as composite according to FusedTriplets (x-axis) and with at least two known domains according to Pfam (A) and CDD (B) (y-axis). Each point corresponds to a family. Since these points can stack, isodensity lines in blue delimit regions having constant density. (PNG 2568 kb)

Additional file 3: **Figure S3.** Alternative phylogeny of eukaryotes, based on Burki et al. [84], that shows the distribution of 573 S-gene families. Family evolution reconstruction was performed using Dollo parsimony. The four boxes correspond to the number of families involved in metabolism (red), information storage and processing (blue), cellular processes and signaling (green), and poorly characterized processes (white). Few families are found in the internal node of Archaeplastida and Cryptophyta (seven families) and in the internal node of SAR and Haptophyta (four families). (PNG 585 kb)

Additional file 4: **Figure S4.** Putative phylogeny of eukaryotes, based on He et al. [85], that shows the distribution of 573 S-gene families. Family evolution reconstruction was performed using Dollo parsimony. The four boxes correspond to the number of families involved in metabolism (red), information storage and processing (blue), cellular processes and signaling (green), and poorly characterized processes (white). This tree topology decreases dramatically the number of early families (152 families). However, this change is largely dependent on the unbalanced distribution of genomes between Discoba (only three genomes) and the Opimoda + Diphoda group (35 genomes). (PNG 594 kb)

Additional file 5: Results of phylogenetic analysis of S-gene families and of components of S-genes. (XLSX 78 kb)

Additional file 6: Annotation of the 573 S-gene families detected in our study. Columns B, C, and D correspond to the EggNog automatic annotation. Column K corresponds to the manual annotation. Columns H, I, and J correspond to additional annotations for S-gene families present in the well-annotated model organism *Saccharomyces cerevisiae* (gene symbol, gene essentiality, and protein complexes). Columns L and M show the most abundant common protein architecture according CDD and Pfam (numbers between brackets correspond to the percentage of proteins in the given family having the given protein architecture). Column N corresponds to the cluster assignment found in Fig. 4, while columns O, P, Q, and R correspond to the ratios used to determine these clusters. Column S corresponds to the consistency between BLASTP and phylogeny strategies for taxonomic assignment. Column T corresponds to the domains removed for the ‘conservative’ component origin assignment and columns U, V, W, X, and Y correspond to the cluster and the ratios computed for the conservative assignment. The column Z indicates families for which the detection of components is restricted (restricted) to a portion of the S-gene (i.e., BAC-X/ARC-X/PROK-X). Column Z also indicates the families carrying BAC/ARC/PROK components detected by HMM (HMM-detected-component). Columns AA, AB, AC, AD, and AE correspond to the subcellular localization performed using TargetP. Columns AA, AB, AC, and AD correspond to the ratio of protein members having a mitochondrion transit peptide, chloroplast transit peptide, a signal peptide, and any other location, respectively. Column AE is the general annotation regarding whether the family is targeted or not (if more than 50% of the members of a family were predicted to contain a signal or a transit peptide, the family was considered as targeted). Columns AF and AG correspond to information about intron conservation within and between components, respectively. (XLSX 208 kb)

Additional file 7: **Figure S5.** One example of intron position conservation between one Opimoda (*M. brevicollis*, gi: 167536479) and one Diphoda (*P. tetraurelia*, gi:145550193) S-genes (family 11,734). Each sequence is represented in red. The yellow circles represent the intron positions on the sequence. The black segments on top of each sequence show the component positions. Their position is also mapped on the S-genes in blue. Each conserved intron is numbered. The conserved introns localized between two components are in orange. (PNG 263 kb)

Additional file 8: **Figure S6.** Distribution of 573 S-gene families across eukaryotic species. The heatmap represents the presence (black line) or absence (white line) of a given S-gene family in a eukaryotic species (each line represents a given species, each column represents a given family). Eukaryotic species are colored with respect to their classification into major supergroups (light green: Archaeaplastida, dark yellow: Cryptophytes, yellow: Haptophytes, light blue: Rhizaria, blue: Alveolates, purple: Stramenopiles, brown: excavates, red: Opisthokonts, orange: Amoebozoa). The colored top bar indicates the functional annotation of the S-gene families according to COG (red: metabolism, blue: information storage and processing, green: cellular processes and signaling, white: poorly characterized). The heatmap is structured along its x-axis, based on the number of eukaryotic supergroups containing the S-gene family, binned in decreasing order (from the left: S-gene families distributed in all nine supergroups, to the right: S-gene families present in a single supergroup but in at least three species; each bin is separated by a thin red line). (PNG 638 kb)

Additional file 9: **Figure S7.** KEGG map of the spliceosome showing the 15 S-genes in green (4504: Prp19, 3721; U1A/U2B”, 6543: Prp17, 5353: SF3b, 16,534: SF3b, 60,389: SF3b, 14,116: U2AF, 39,809: PUF60, 20,969: SR140, 7536: Prp31, 3214: Brr2, 4638: Syf, 8301: RBM22, 7062: AQR, 60753: SR). (PNG 47 kb)

Additional file 10: **Figure S8.** KEGG map of the glycerophospholipid pathway showing the 6 S-genes (26,775: 1.1.1.8; 19,545: 2.3.1.42; 60,473: 2.7.1.107; 5156: 2.7.7.14; 30,146: 3.1.4.4, 26,228: LPGAT/LPCAT, 26810: BTA1). (PNG 42 kb)

Additional file 11: **Figure S9.** Protocol used for the taxonomic assignment of S-gene families. A. For each component of S-proteins, taxonomic assignment was performed based on the 10 best BLASTP hits. B. Taxonomic assignment information was summed up at the family level, each S-gene family received a ratio that represented the proportion of the components with a given origin (ARC, BAC, PROK, EUK). These values were then used to cluster families having similar component origins (Fig. 4). (PNG 480 kb)

Additional file 12: Detailed origin of prokaryotic S-gene components. Fam: S-gene family, Cpt: component, Cluster: cluster number according to Fig. 4, Bacteria: number of hits from Bacteria, Archaea: number of hits from Archaea. For each S-gene component, the rank in the BLAST search and the taxonomic assignation of the 25 sequences with the best hits to that component were reported (Aci: *Acidobacteria*, Act: *Actinobacteria*, Aqu: *Aquificae*, Arm: *Armatimonadetes*, Bac: *Bacteroidetes*, Chl: *Chloroflexi*, Cre: *Crenarchaeota*, Cya: *Cyanobacteria*, Def: *Deferribacteres*, Dei: *Deinococcus-Thermus*, Eur: *Euryarchaeota*, Fir: *Firmicutes*, Fus: *Fusobacteria*, Gem: *Gemmatimonadetes*, Ign: *Ignavibacteriae*, Nit: *Nitrospirae*, Pla: *Planctomycetes*, Pro: *Proteobacteria*, Spi: *Spirochaetes*, Syn: *Synergistetes*, Ten: *Tenericutes*, Tha: *Thaumarchaeota*, The: *Thermotogae*, Ver: *Verrucomicrobia*, roo: Unknown). Red cells correspond to bacterial phyla while blue cells correspond to archaeal phyla. When only one S-gene component is described, the unrepresented S-gene components from the S-gene family are either exclusively found in photosynthetic eukaryotes, or have diverged too much to be confidently assigned to a prokaryotic group. (XLSX 1743 kb)

Additional file 13: **Figure S10.** S-gene family 18,563. A. Component architecture and phylogenetic tree of S-gene family 18,563. Family 18,563 is composed of one component (RNA methyltransferase (MTase)) of archaeal origin according to our BLASTp taxonomic assignment and two domains of eukaryotic origins (for the phylogenetic tree, blue: SAR, red: Archaeplastida, purple: Opisthokonta, cyan: Haptophyta, orange: Amoebozoa, blue-green: Discoba) (13 sequences, 599 sites, model LG + I + G4, 1000 ultrafast bootstraps). B. ML phylogenetic tree of the MTase component (green: Eukarya, blue: Archaea, red: Bacteria, black circle: bootstraps > 80%) (113 sequences, 146 sites, model LG + I + G4, 1000 ultrafast bootstraps). (PNG 2662 kb)

Additional file 14: **Figure S11.** S-gene family 6384. A. Component architecture and phylogenetic tree of S-gene family 6384. Family 6384 is composed of one component (glutathione S-transferase (GST)) of bacterial origin according to our BLASTp taxonomic assignment and of two domains of eukaryotic origins (for the phylogenetic tree, blue: SAR, red: Archaeplastida, purple: Opisthokonta, orange: Amoebozoa) (27 sequences, 315 sites, model LG + I + G4, 1000 ultrafast bootstraps). B. ML phylogenetic tree of the GST component (green: Eukarya, blue: Archaea, red: Bacteria, black circle: bootstraps > 80%) (127 sequences, 172 sites, model LG + I + G4, 1000 ultrafast bootstraps). (PNG 2925 kb)

Additional file 15: **Figure S12.** S-gene family 9304. A. Component architecture and phylogenetic tree of S-gene family 9304. Family 9304 is composed of two components (ATP-citrate lyase subunits A and B (ACLA and ACLB)) of prokaryotic origin according to our BLASTp taxonomic assignment (for the phylogenetic tree, blue: SAR, orange: Amoebozoa, purple: Opisthokonta) (15 sequences, 1171 sites, model LG + I + G4, 1000 ultrafast bootstraps). B. Maximum-likelihood phylogenetic tree of the ACLA component (green: Eukarya, blue: Archaea, red: Bacteria, black circle: bootstraps > 80%) (115 sequences, 364 sites, model LG + I + G4, 1000 ultrafast bootstraps). C. Maximum-likelihood phylogenetic tree of the ACLB component (green: Eukarya, blue: Archaea, red: Bacteria, black circle: bootstraps > 80%) (115 sequences, 485 sites, model LG + I + G4, 1000 ultrafast bootstraps). (PNG 3389 kb)

Additional file 16: **Figure S13.** χ^2^ test of the distribution of COG categories. The color code is the same as in Fig. 4. Barplots correspond to observed proportions while black lines correspond to expected proportions (ARC-X: clusters 6, 8, 9 and 10; BAC-BAC: clusters 2 and 7, BAC-EUK: cluster 3, PROK-BAC: cluster 5, PROK-PROK: cluster 4, and PROK-EUK: cluster 1 in Fig. 4). (PNG 466 kb)

Additional file 17: **Figure S14.** Functional annotation of the S-genes involved in information storage and processing according to the different clusters in Fig. 4 (ARC-X: clusters 6, 8, 9, and 10; BAC-BAC: clusters 2 and 7, BAC-EUK: cluster 3, PROK-BAC: cluster 5, PROK-PROK: cluster 4 and PROK-EUK: cluster 1). (PNG 365 kb)

Additional file 18: **Figure S15.** Hierarchical clustering of S-gene families according to their component origins using the conservative taxonomic assignment. The heatmap represents the ratio of genes in a given family (columns) that have at least one component of a given origin (eukaryotic, archaeal, bacterial or prokaryotic; the rows). White lines correspond to the absence of a component from a given origin in every gene in the given S-gene family. The colored lines correspond to the presence of at least one component of the given origin in a given percentage of genes in the given S-gene family (red lines denote that all (100%) genes contain a given origin component). The first colored top bar indicates the functional annotation. The black bars in the second colored top bar indicate the reclassified S-genes after applying the HMM-profile procedure. Cluster 1 roughly corresponds to 103 S-genes with bacterial and eukaryotic components (BAC-EUK), cluster 2 roughly corresponds to 67 S-genes with prokaryotic and bacterial components (PROK-EUK), cluster 3 roughly corresponds to 139 S-genes with only bacterial components (BAC-BAC), cluster 4 roughly corresponds to 119 S-genes with prokaryotic and eukaryotic components (PROK-EUK), cluster 5 roughly corresponds to 84 S-genes with only prokaryotic components (PROK-PROK), cluster 6 roughly corresponds to 21 S-genes with archaeal and eukaryotic components (ARC-EUK), cluster 7 roughly corresponds to a mix of 11 S-genes with only archaeal components (ARC-ARC) and with archaeal and prokaryotic components (ARC-PROK), and finally cluster 8 roughly corresponds to 7 S-genes with bacterial and archaeal components (ARC-BAC). (PNG 904 kb)

Additional file 19: **Figure S16.** χ^2^ test of the distribution of COG categories (conservative taxonomic assignment). The color code is the same as in Additional file 15: Figure S12. Barplots correspond to observed proportions while black lines correspond to expected proportions (ARC-X: clusters 6, 7, 8, BAC-BAC: cluster 3, BAC-EUK: cluster 1, PROK-BAC: cluster 2, PROK-PROK: cluster 5 and PROK-EUK: cluster 4 in Additional file 15: Figure S12). (PNG 327 kb)

Additional file 20: List of 38 eukaryote genomes and the 382 prokaryotic genomes used in our comparative analysis. (XLSX 277 kb)

Additional file 21: Supporting data for this study, consisting of fasta sequences of S-genes, intron position alignments, components information, and phylogenetic trees. (ZIP 11370 kb)
