# Supplementary figures and images for: Formation of chimeric genes with essential functions at the origin of eukaryotes
Source: BMC Biol. 2018 Mar 13;16:30. doi: 10.1186/s12915-018-0500-0 (PMC5851275; doi:10.1186/s12915-018-0500-0)

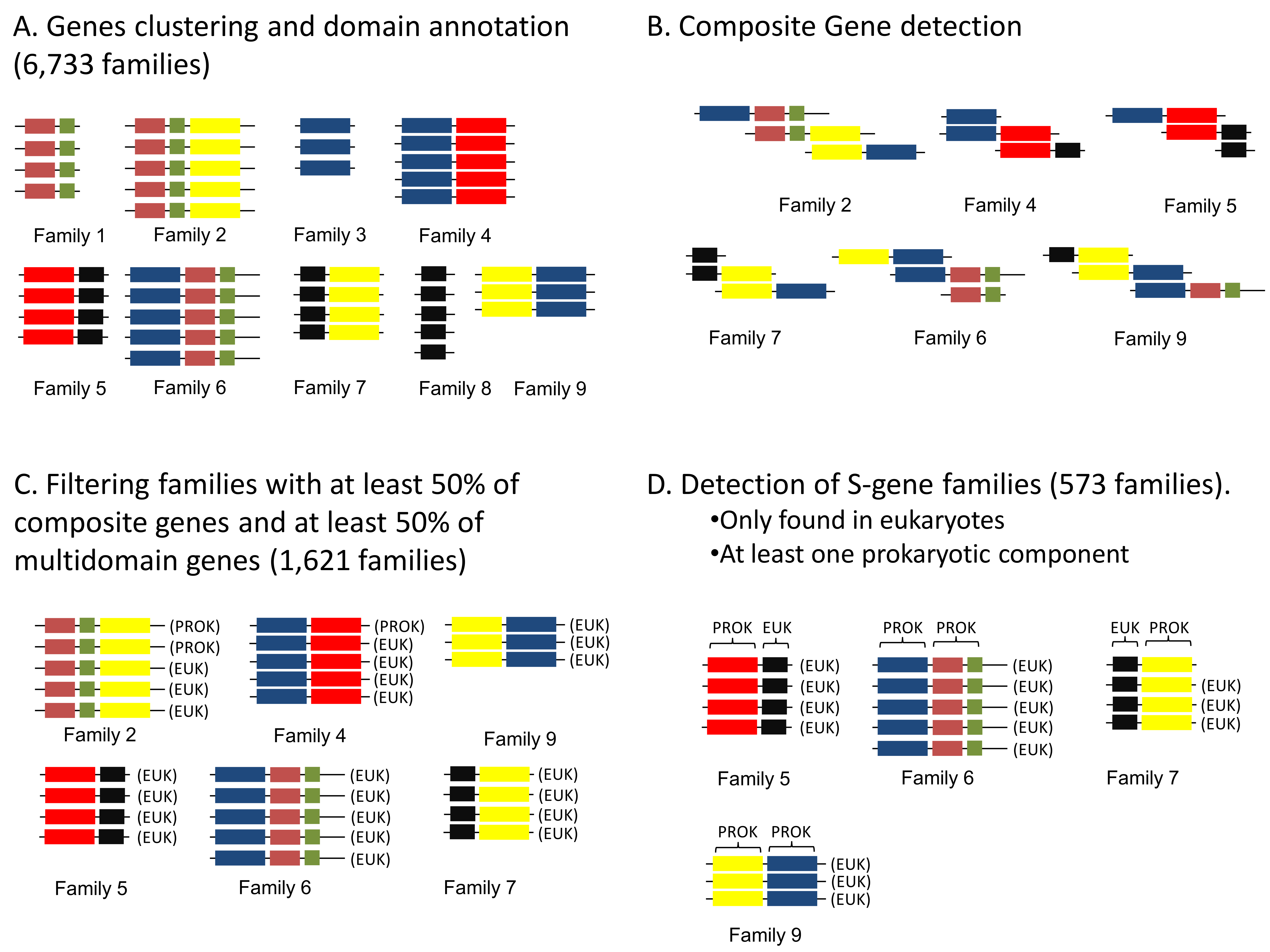

Supplement: Supplementary file 1 — Figure S1. Protocol used for the detection of S-gene families. A. Sequences have been clustered in gene families. B. Composite genes have been detected using FusedTriplets. C. Gene families detected as composite and having at least two domains have been kept for further analysis. D. Composite gene families only found in eukaryotes and having at least one component of prokaryotic origin were considered as S-gene families. (PNG 970 kb) [file 12915_2018_500_MOESM1_ESM.png]

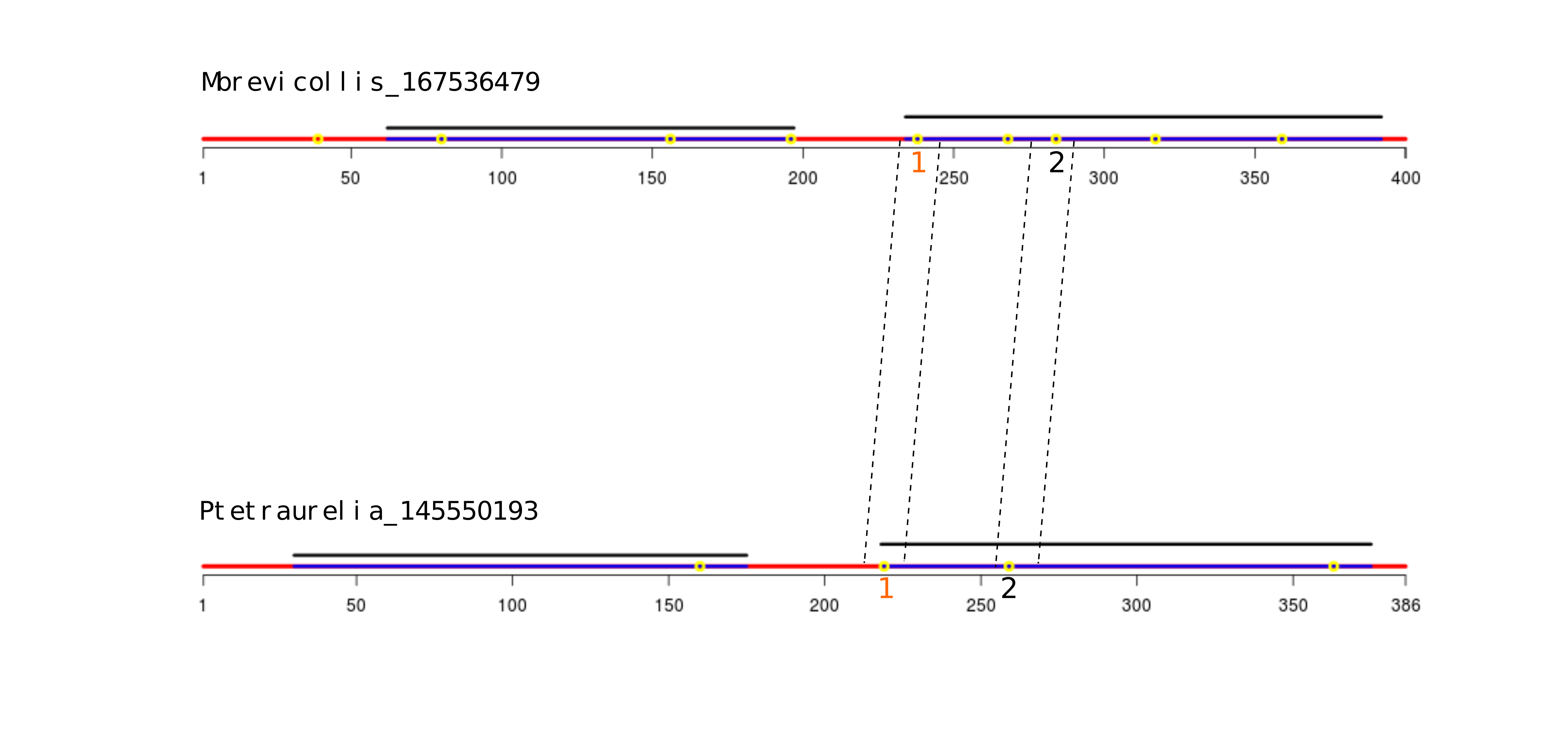

Supplement: Supplementary file 7 — Figure S5. One example of intron position conservation between one Opimoda (M. brevicollis, gi: 167536479) and one Diphoda (P. tetraurelia, gi:145550193) S-genes (family 11,734). Each sequence is represented in red. The yellow circles represent the intron positions on the sequence. The black segments on top of each sequence show the component positions. Their position is also mapped on the S-genes in blue. Each conserved intron is numbered. The conserved introns localized between two components are in orange. (PNG 263 kb) [file 12915_2018_500_MOESM7_ESM.png]

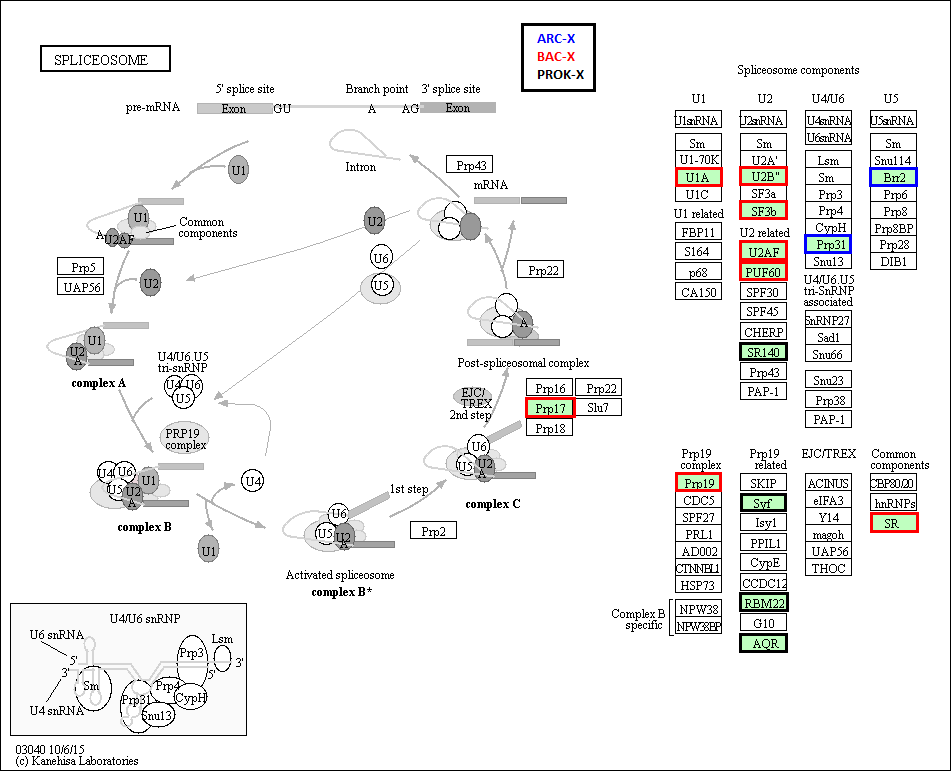

Supplement: Supplementary file 9 — Figure S7. KEGG map of the spliceosome showing the 15 S-genes in green (4504: Prp19, 3721; U1A/U2B”, 6543: Prp17, 5353: SF3b, 16,534: SF3b, 60,389: SF3b, 14,116: U2AF, 39,809: PUF60, 20,969: SR140, 7536: Prp31, 3214: Brr2, 4638: Syf, 8301: RBM22, 7062: AQR, 60753: SR). (PNG 47 kb) [file 12915_2018_500_MOESM9_ESM.png]

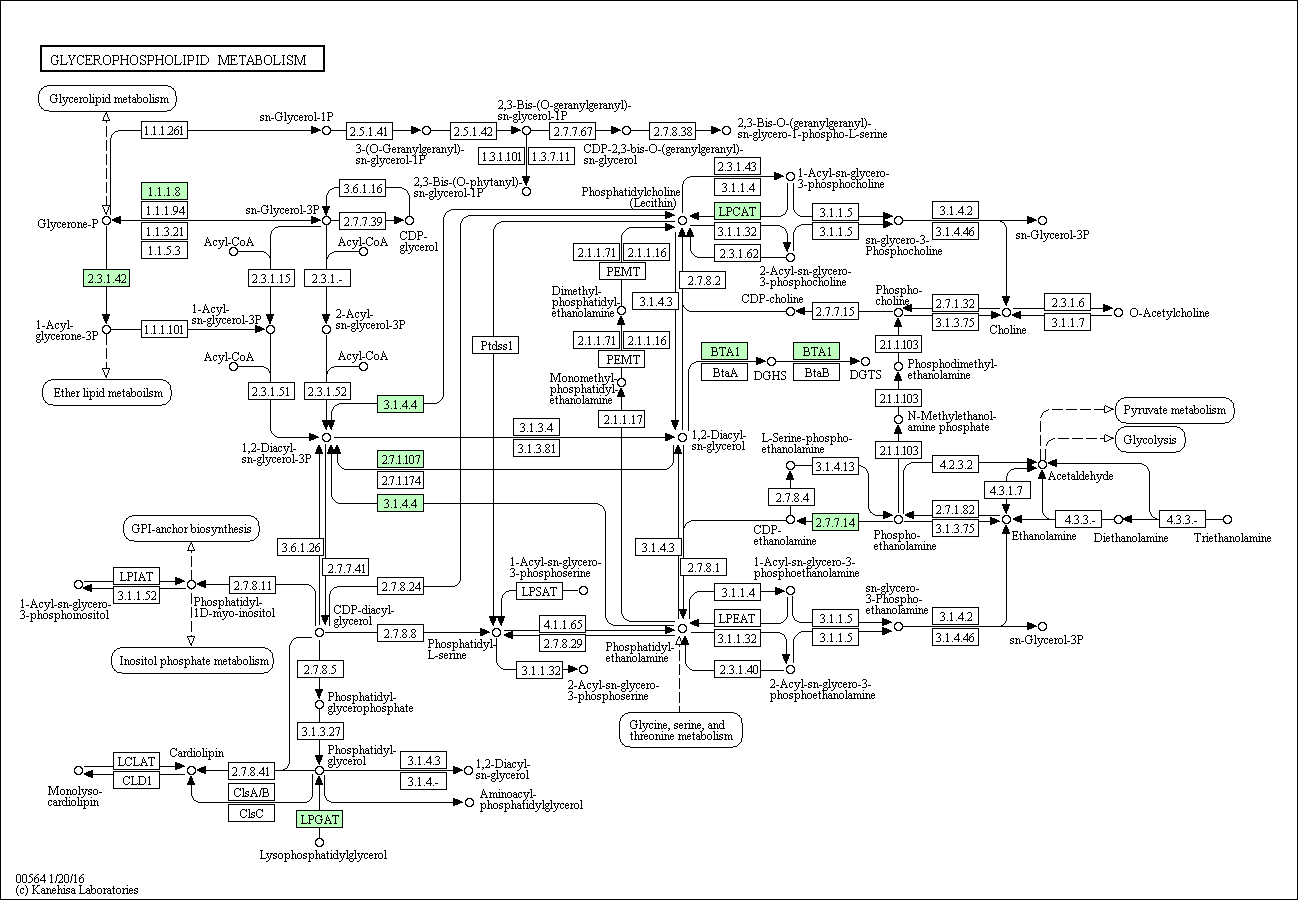

Supplement: Supplementary file 10 — Figure S8. KEGG map of the glycerophospholipid pathway showing the 6 S-genes (26,775: 1.1.1.8; 19,545: 2.3.1.42; 60,473: 2.7.1.107; 5156: 2.7.7.14; 30,146: 3.1.4.4, 26,228: LPGAT/LPCAT, 26810: BTA1). (PNG 42 kb) [file 12915_2018_500_MOESM10_ESM.png]

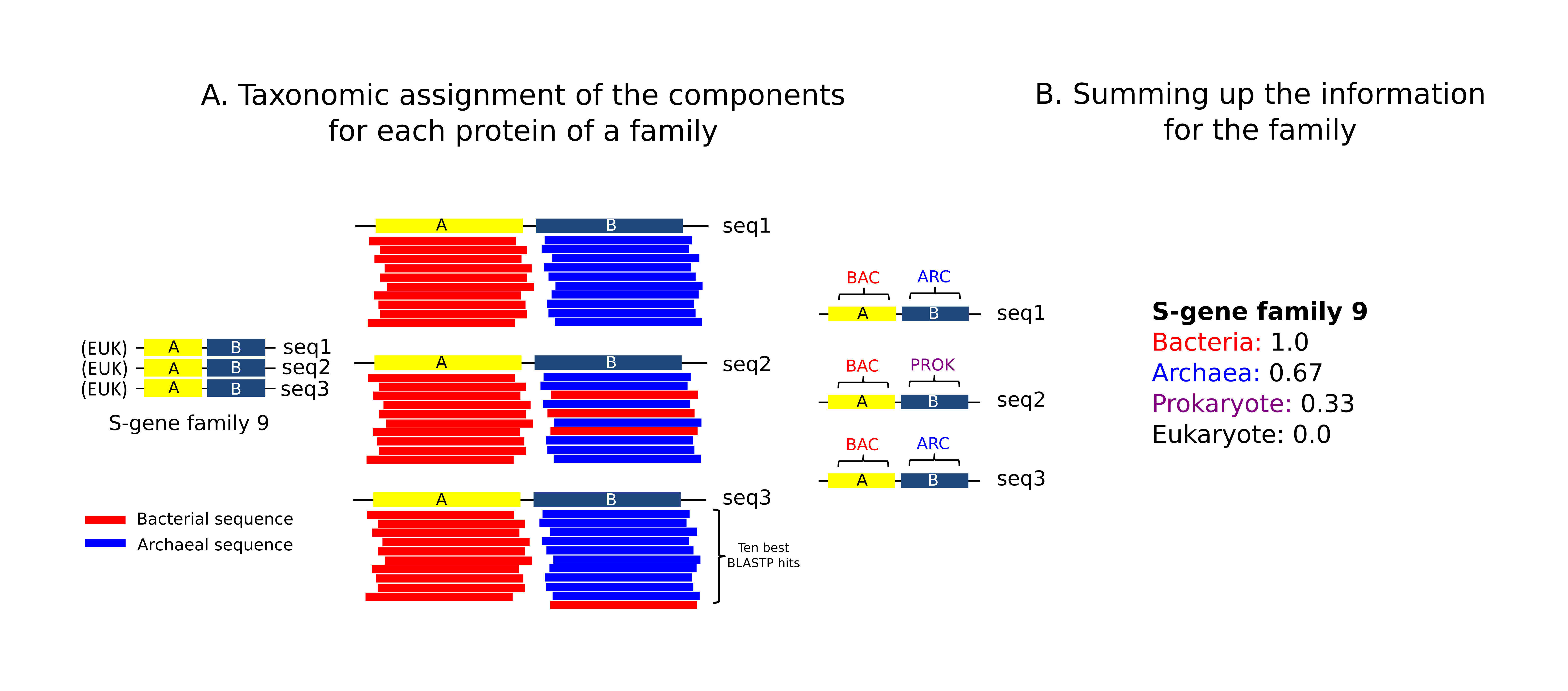

Supplement: Supplementary file 11 — Figure S9. Protocol used for the taxonomic assignment of S-gene families. A. For each component of S-proteins, taxonomic assignment was performed based on the 10 best BLASTP hits. B. Taxonomic assignment information was summed up at the family level, each S-gene family received a ratio that represented the proportion of the components with a given origin (ARC, BAC, PROK, EUK). These values were then used to cluster families having similar component origins (Fig. 4). (PNG 480 kb) [file 12915_2018_500_MOESM11_ESM.png]

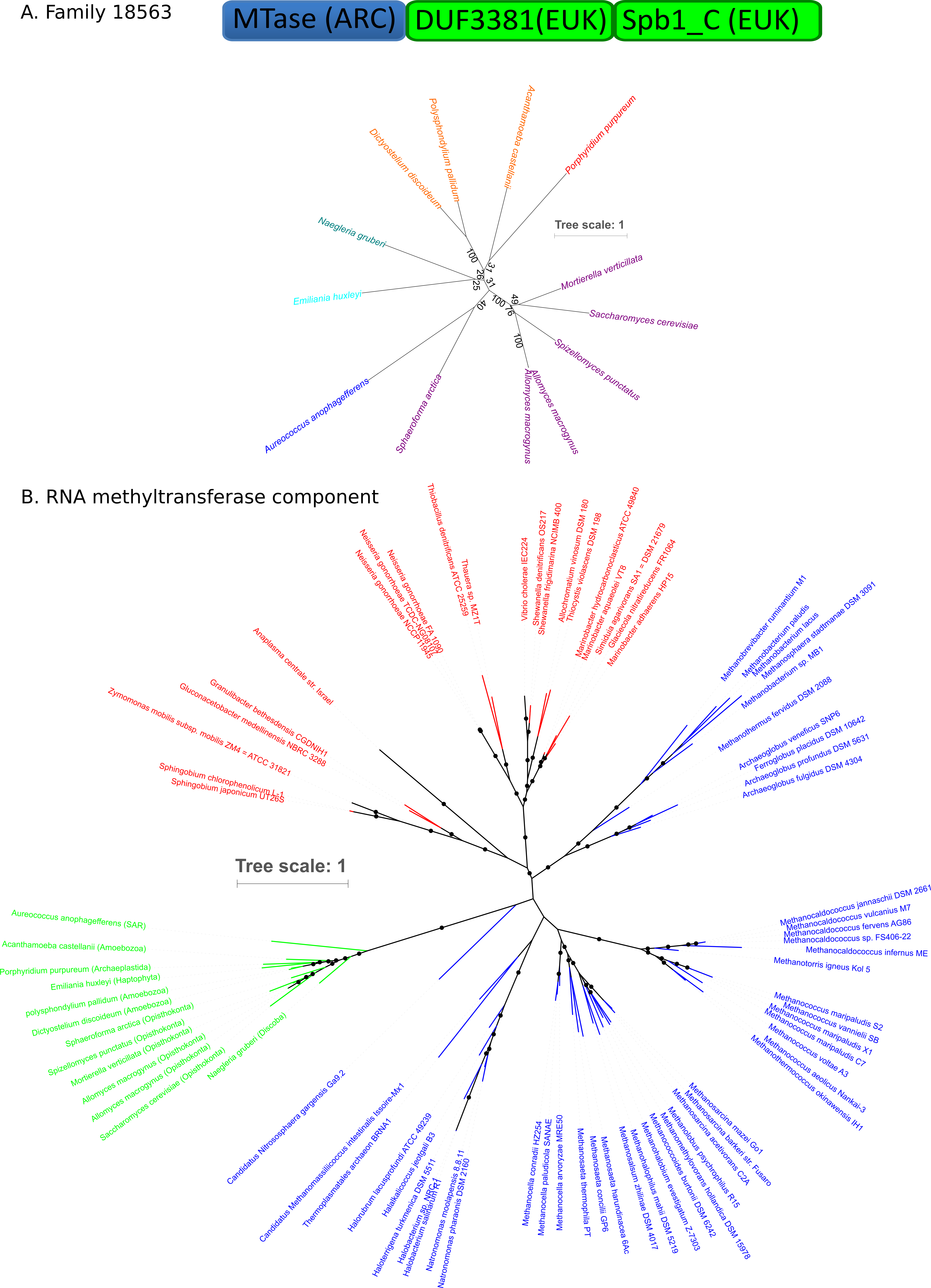

Supplement: Supplementary file 13 — Figure S10. S-gene family 18,563. A. Component architecture and phylogenetic tree of S-gene family 18,563. Family 18,563 is composed of one component (RNA methyltransferase (MTase)) of archaeal origin according to our BLASTp taxonomic assignment and two domains of eukaryotic origins (for the phylogenetic tree, blue: SAR, red: Archaeplastida, purple: Opisthokonta, cyan: Haptophyta, orange: Amoebozoa, blue-green: Discoba) (13 sequences, 599 sites, model LG + I + G4, 1000 ultrafast bootstraps). B. ML phylogenetic tree of the MTase component (green: Eukarya, blue: Archaea, red: Bacteria, black circle: bootstraps > 80%) (113 sequences, 146 sites, model LG + I + G4, 1000 ultrafast bootstraps). (PNG 2662 kb) [file 12915_2018_500_MOESM13_ESM.png]

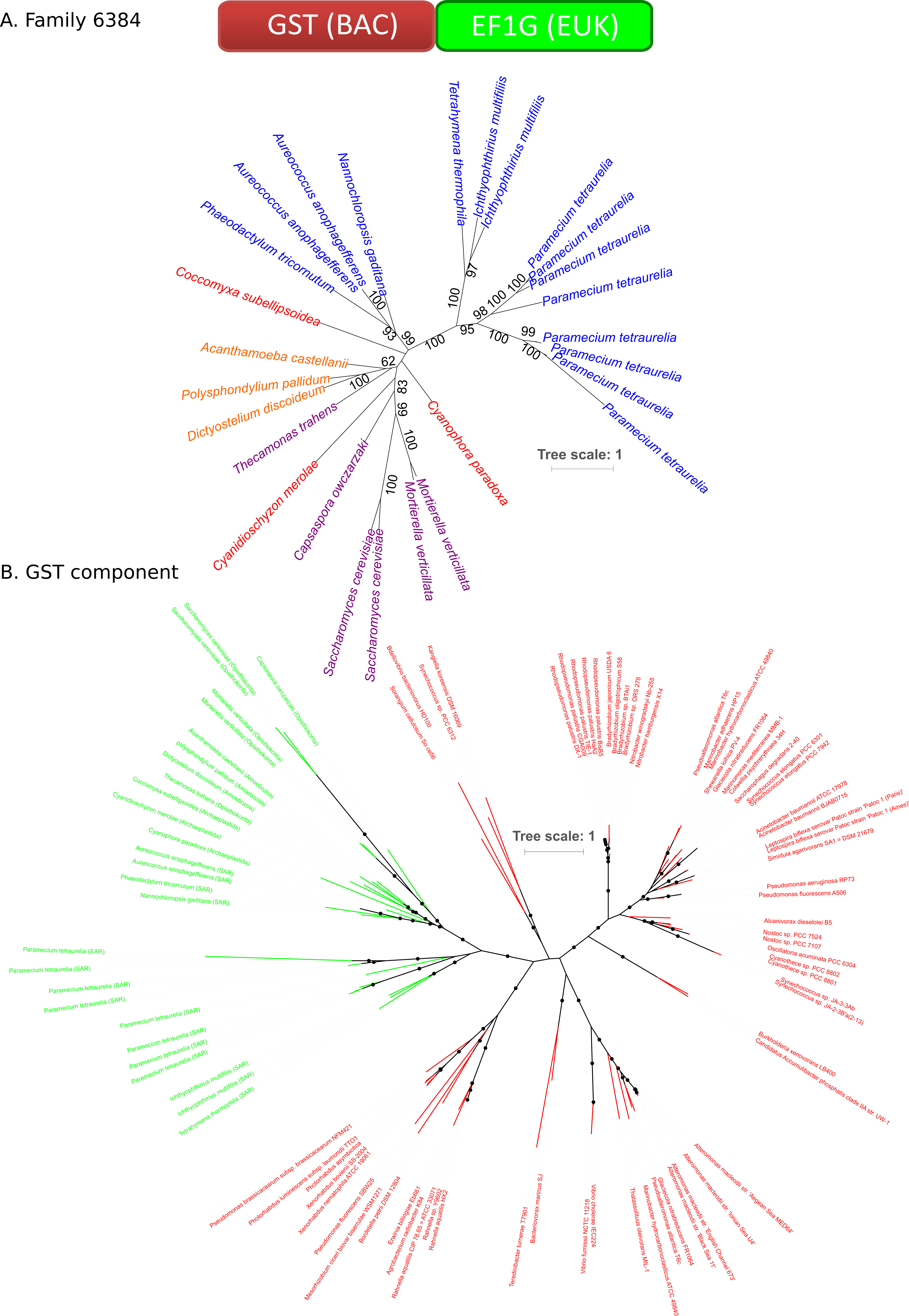

Supplement: Supplementary file 14 — Figure S11. S-gene family 6384. A. Component architecture and phylogenetic tree of S-gene family 6384. Family 6384 is composed of one component (glutathione S-transferase (GST)) of bacterial origin according to our BLASTp taxonomic assignment and of two domains of eukaryotic origins (for the phylogenetic tree, blue: SAR, red: Archaeplastida, purple: Opisthokonta, orange: Amoebozoa) (27 sequences, 315 sites, model LG + I + G4, 1000 ultrafast bootstraps). B. ML phylogenetic tree of the GST component (green: Eukarya, blue: Archaea, red: Bacteria, black circle: bootstraps > 80%) (127 sequences, 172 sites, model LG + I + G4, 1000 ultrafast bootstraps). (PNG 2925 kb) [file 12915_2018_500_MOESM14_ESM.png]

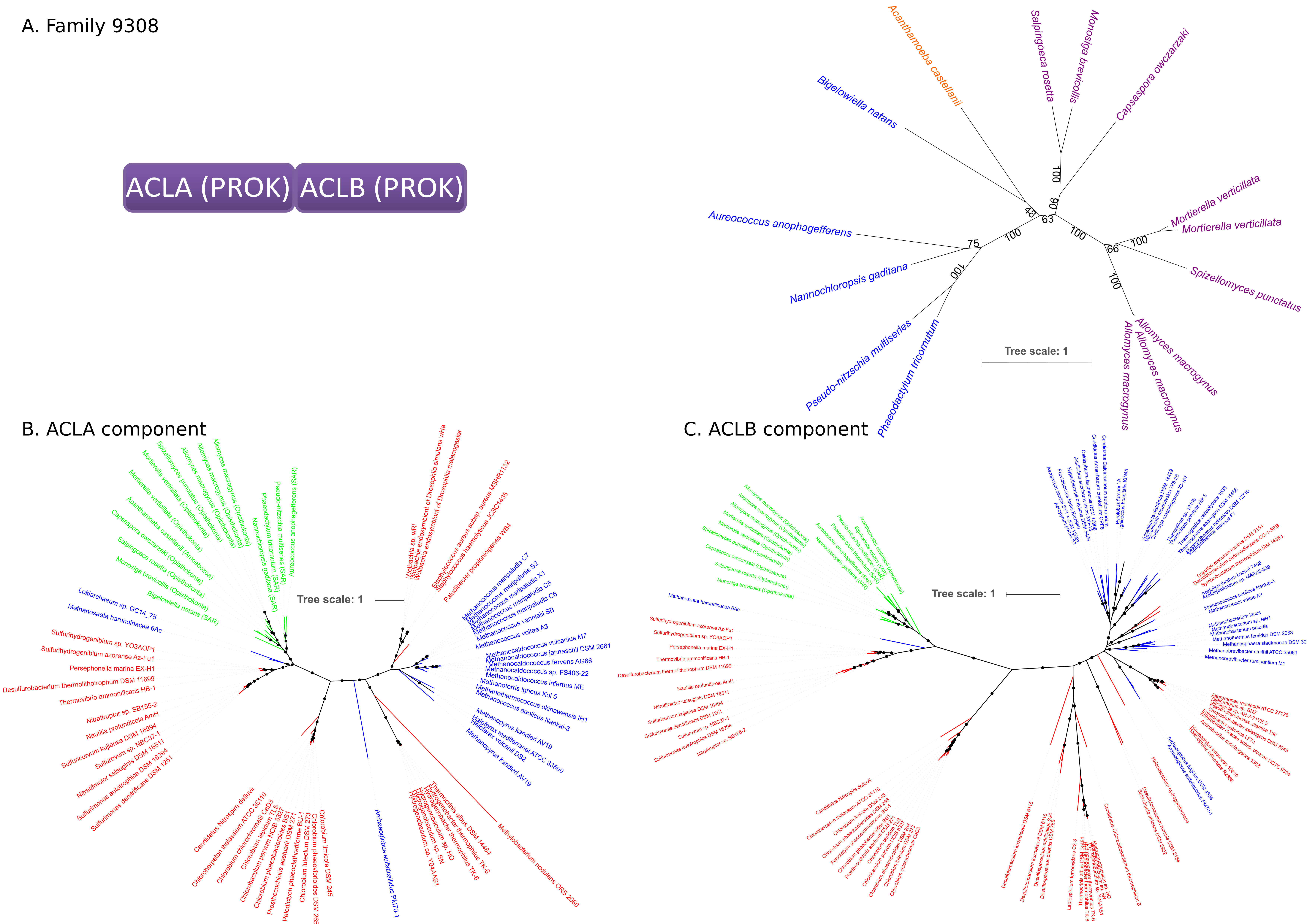

Supplement: Supplementary file 15 — Figure S12. S-gene family 9304. A. Component architecture and phylogenetic tree of S-gene family 9304. Family 9304 is composed of two components (ATP-citrate lyase subunits A and B (ACLA and ACLB)) of prokaryotic origin according to our BLASTp taxonomic assignment (for the phylogenetic tree, blue: SAR, orange: Amoebozoa, purple: Opisthokonta) (15 sequences, 1171 sites, model LG + I + G4, 1000 ultrafast bootstraps). B. Maximum-likelihood phylogenetic tree of the ACLA component (green: Eukarya, blue: Archaea, red: Bacteria, black circle: bootstraps > 80%) (115 sequences, 364 sites, model LG + I + G4, 1000 ultrafast bootstraps). C. Maximum-likelihood phylogenetic tree of the ACLB component (green: Eukarya, blue: Archaea, red: Bacteria, black circle: bootstraps > 80%) (115 sequences, 485 sites, model LG + I + G4, 1000 ultrafast bootstraps). (PNG 3389 kb) [file 12915_2018_500_MOESM15_ESM.png]

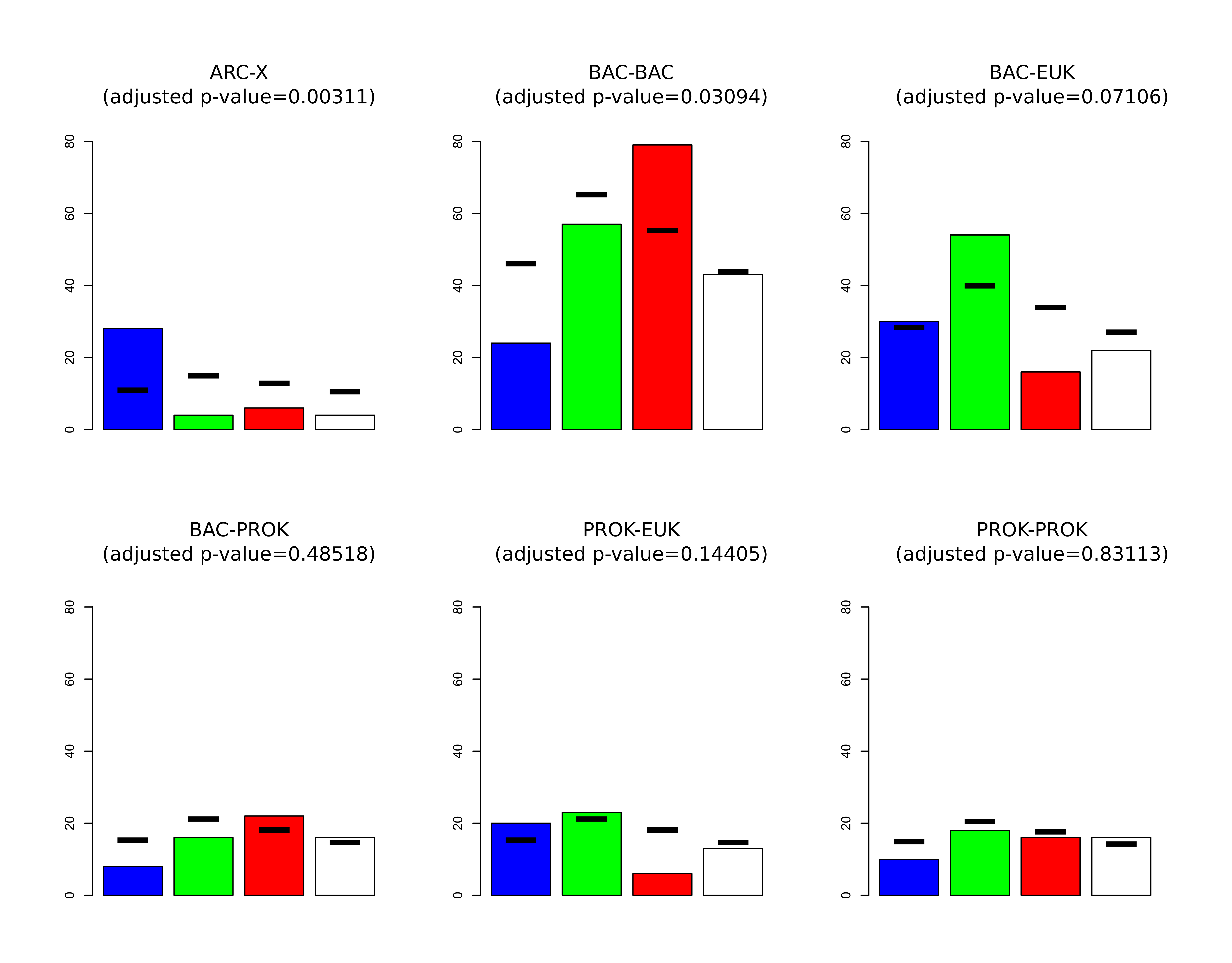

Supplement: Supplementary file 16 — Figure S13. χ2 test of the distribution of COG categories. The color code is the same as in Fig. 4. Barplots correspond to observed proportions while black lines correspond to expected proportions (ARC-X: clusters 6, 8, 9 and 10; BAC-BAC: clusters 2 and 7, BAC-EUK: cluster 3, PROK-BAC: cluster 5, PROK-PROK: cluster 4, and PROK-EUK: cluster 1 in Fig. 4). (PNG 466 kb) [file 12915_2018_500_MOESM16_ESM.png]

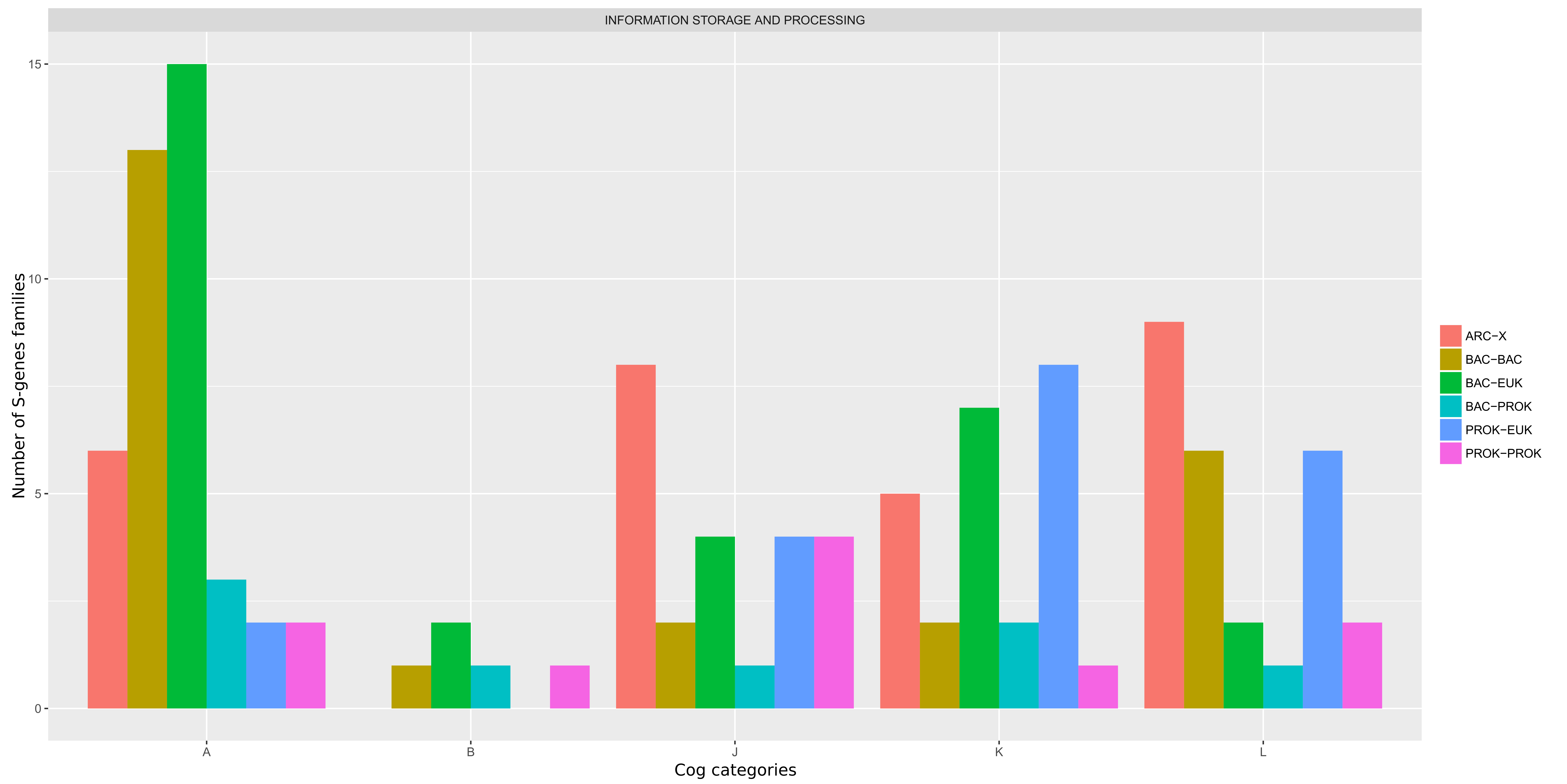

Supplement: Supplementary file 17 — Figure S14. Functional annotation of the S-genes involved in information storage and processing according to the different clusters in Fig. 4 (ARC-X: clusters 6, 8, 9, and 10; BAC-BAC: clusters 2 and 7, BAC-EUK: cluster 3, PROK-BAC: cluster 5, PROK-PROK: cluster 4 and PROK-EUK: cluster 1). (PNG 365 kb) [file 12915_2018_500_MOESM17_ESM.png]

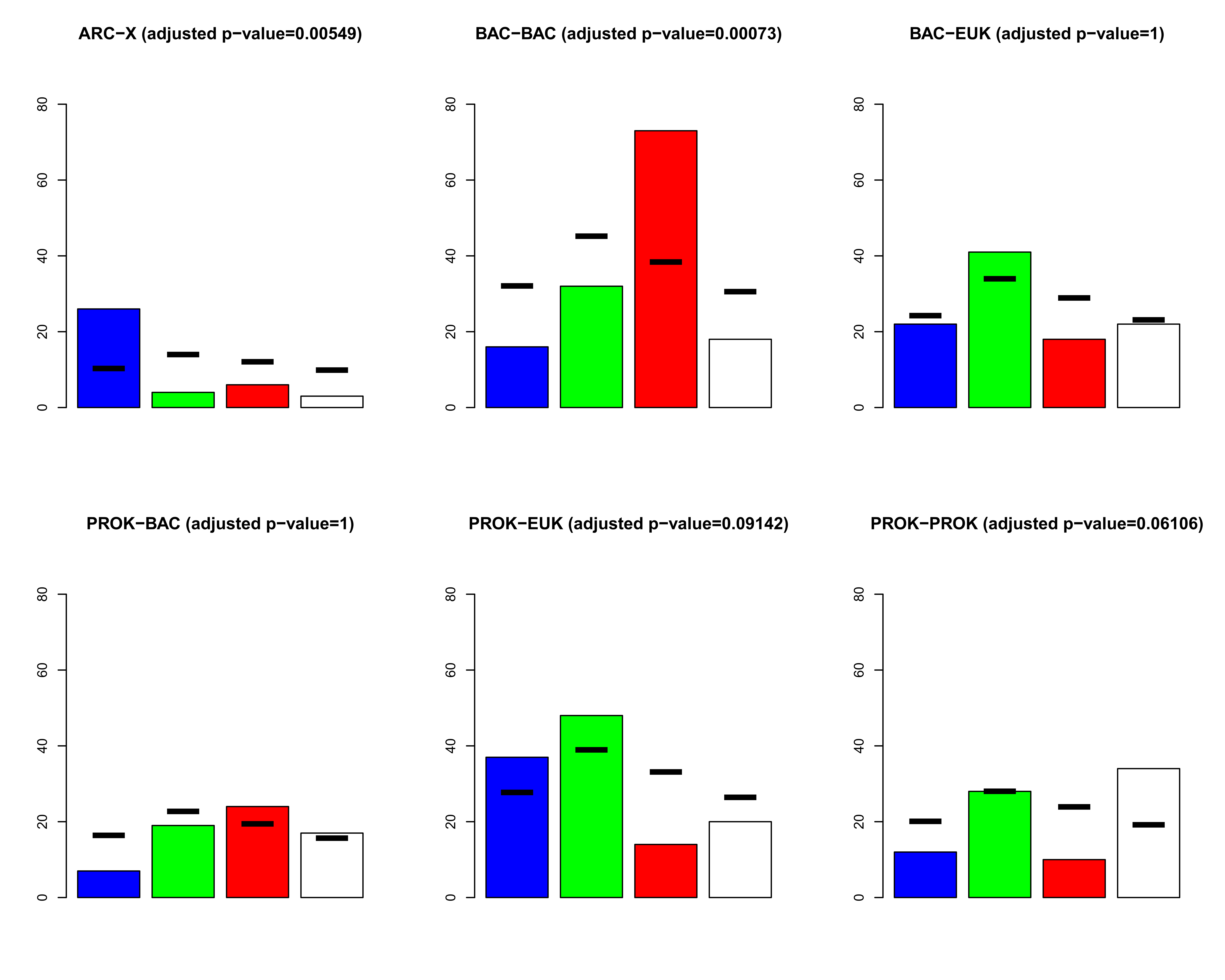

Supplement: Supplementary file 19 — Figure S16. χ2 test of the distribution of COG categories (conservative taxonomic assignment). The color code is the same as in Additional file 15: Figure S12. Barplots correspond to observed proportions while black lines correspond to expected proportions (ARC-X: clusters 6, 7, 8, BAC-BAC: cluster 3, BAC-EUK: cluster 1, PROK-BAC: cluster 2, PROK-PROK: cluster 5 and PROK-EUK: cluster 4 in Additional file 15: Figure S12). (PNG 327 kb) [file 12915_2018_500_MOESM19_ESM.png]
